# Ubiquitination of stalled ribosome triggers ribosome-associated quality control

**DOI:** 10.1038/s41467-017-00188-1

**Published:** 2017-07-31

**Authors:** Yoshitaka Matsuo, Ken Ikeuchi, Yasushi Saeki, Shintaro Iwasaki, Christian Schmidt, Tsuyoshi Udagawa, Fumiya Sato, Hikaru Tsuchiya, Thomas Becker, Keiji Tanaka, Nicholas T. Ingolia, Roland Beckmann, Toshifumi Inada

**Affiliations:** 1 Graduate School of Pharmaceutical Sciences, Tohoku University, Sendai, 980-8578 Japan; 2 Laboratory of Protein Metabolism, Tokyo Metropolitan Institute of Medical Science, Setagaya-ku, Tokyo 156-8506 Japan; 3 Department of Molecular and Cell Biology, University of California, Berkeley, CA 94720 USA; 4 Gene Center and Center for Integrated Protein Science Munich, Department of Biochemistry, University of Munich, Feodor-Lynen-Str. 25, Munich, 81377 Germany; 5 Present Address: RIKEN, Saitama, 351-0198 Japan

## Abstract

Translation arrest by polybasic sequences induces ribosome stalling, and the arrest product is degraded by the ribosome-mediated quality control (RQC) system. Here we report that ubiquitination of the 40S ribosomal protein uS10 by the E3 ubiquitin ligase Hel2 (or RQT1) is required for RQC. We identify a RQC-trigger (RQT) subcomplex composed of the RNA helicase-family protein Slh1/Rqt2, the ubiquitin-binding protein Cue3/Rqt3, and yKR023W/Rqt4 that is required for RQC. The defects in RQC of the RQT mutants correlate with sensitivity to anisomycin, which stalls ribosome at the rotated form. Cryo-electron microscopy analysis reveals that Hel2-bound ribosome are dominantly the rotated form with hybrid tRNAs. Ribosome profiling reveals that ribosomes stalled at the rotated state with specific pairs of codons at P-A sites serve as RQC substrates. Rqt1 specifically ubiquitinates these arrested ribosomes to target them to the RQT complex, allowing subsequent RQC reactions including dissociation of the stalled ribosome into subunits.

## Abstract (web summary)

Several protein quality control mechanisms are in place to trigger the rapid degradation of aberrant polypeptides and mRNAs. Here the authors describe a mechanism of ribosome-mediated quality control that involves the ubiquitination of ribosomal proteins by the E3 ubiquitin ligase Hel2/RQT1.

## Introduction

Accurate gene expression is a prerequisite for all cellular processes. Quality control machineries respond to abnormal translational elongation and termination, which result in the rapid degradation of aberrant polypeptides and mRNAs. Translation arrest results in the degradation of the arrest products by the ribosome-mediated quality control system (RQC)^1–4^. In the RQC pathway, the subunit dissociation is a key step for an E3 ubiquitin ligase Ltn1-dependent ubiquitination of the arrest products on the 60S large ribosomal subunit^5–8^, leading to subsequent proteasomal degradation^1, 2, 9–12^.

Aberrant translation arrest induced by poly-lysine sequence results in RQC in yeast^1, 2, 4, 13–20^, in *Droshophila* ^21, 22^, and in mammalian cells^8, 9, 23–26^. The yeast Ltn1 was identified by genetic screen as a factor that is involved in the repression of aberrant nonstop products with poly-lysine sequence produced by translation of a poly(A) tail of nonstop mRNA^27^. A mouse forward genetics screen identified Listerin, mammalian homolog of Ltn1, as a factor involved in neurodegeneration^28^. A search for genetic interactions with Ltn1 and mRNA decay pathways uncovered Rqc1 and Rqc2 in yeast^2, 4, 19^. These factors together comprise the 60S-associated RQC machinery that is required for the efficient degradation of stalled protein products. Ltn1 and Rqc2 were co-purified with Rqc1, the entire 60S ribosomal subunit, and the AAA+ ATPase Cdc48, along with its cofactors Npl4 and Ufd1^2, 4, 19^. Cryo-electron microscopy (Cryo-EM) analysis of the yeast 60S-RQC structures revealed that Rqc2 occupies a large proportion of the inter-subunit surface of 60S and prevents 40S-subunit reassociation after 80S splitting^10, 12, 29^. Rqc1 and the ubiquitination of nascent polypeptide recruit the Cdc48 complex to the ribosome, which sequesters the ubiquitinated nascent polypeptide from the ribosome and delivers it to the proteasome for degradation.

Recent studies uncovered the crucial role of RQC to prevent the formation of proteotoxic protein aggregates derived from translational stalling in yeast^12, 17, 18^. In *ltn1∆* mutant cells, Rqc2 engages the stalled nascent chain, and recruits tRNAs charged with alanine and threonine, so that the 60S catalyzes the C-terminal addition of alanine and threonine to the nascent chain (a “CAT-tail”) in an unusual reaction that is not dependent on either the 40S subunit or mRNA^12^. The CAT-tail mediates the formation of detergent-resistant aggregates along with the poly-lysine tract present in nonstop proteins^17, 18^. The aggregates sequester multiple cytosolic chaperones, including Sis1, thereby interfering with common protein quality control pathways^17, 18^. Rqc2 is required to induce activation of heat-shock factor 1 (Hsf1) when Ltn1 or Rqc1 is lost^2^. Therefore, aggregates of the products of Rqc2-mediated CAT-tail formation may activate Hsf1 to induce chaperones. The mechanism by which the defects in RQC activate Hsf1 should to be resolved to understand the roles of RQC in protein homeostasis.

Genetic screen identifies two key factors, Asc1 and Hel2 that are required for RQC by poly-lysine or poly-arginine sequences in yeast. Asc1 is a 40S ribosomal subunit-associated protein and required for quality controls induced by stalling ribosome within open reading frame (ORF)^14, 30^. Asc1 also acts as a fail-safe in quality control for stalling ribosome at the 3′ end of nonstop mRNA in the absence of Dom34^31^. Hel2 is an E3 ubiquitin ligase and identified as a factor involved in the poly-ubiquitination and proteasomal-degradation of excess histone proteins^32^. Hel2 is also involved in translation arrest by polybasic amino acids sequences^2^, and K63-linked ubiquitination by Hel2 is proposed to be crucial for translation arrest^16^. Most recent study demonstrated that ZNF598 is a mammalian homolog of Hel2 and ubiquitinates ribosome protein eS10 at K138/139 and uS10 at K4/8 residues thereby stimulating the translation arrest by poly-lysine sequence^33, 34^. However, the defects of lysine to arginine substitutions of eS10 or uS10 at the ubiquitination sites were partial, suggesting that translation arrest depends on ribosome ubiquitination at multiple sites in mammal. More importantly, it is still unknown how the ubiquitinated ribosome is recognized and dissociated into the subunits for Ltn1-dependent ubiquitination of peptidyl-tRNA on 60S subunit.

In this study, we investigated how the ribosome ubiquitination induces RQC in yeast. We demonstrated that RQC requires the ubiquitination of the ribosomal protein uS10 at specific lysine residues by the E3 ubiquitin ligase Hel2 and E2 protein Ubc4, indicating that crucial role of ribosome ubiquitination in RQC is conserved. We identified subcomplex that we referred to RQC-trigger (RQT) complex that is essential for RQC. The RQT complex is composed of three proteins, a RNA helicase-family protein Slh1/Rqt2, ubiquitin-binding protein Cue3/Rqt3, and YKR023W/Rqt4, were associated with Hel2-ribosome complexes and required for the primary steps of RQC. The ubiquitin-binding activity of Rqt3 and ATPase activity of Rqt2 were crucial to trigger RQC. The defects in RQC of the RQT mutants correlated with the sensitivity to anisomycin that stalls ribosome at the rotated form, suggesting that RQT factors rescue the stalled ribosome by the drug. Indeed, ribosome profiling revealed that the stalled ribosome at the rotated state with specific pairs of codons at P-A sites serve as RQC substrates. Cryo-EM analysis revealed that Hel2-bound ribosomes are in a rotated state containing hybrid state A/P- and P/E-tRNAs. These results provide us to propose the model that Hel2 binds to rotated ribosomes leading to the ubiquitination of uS10. Subsequently, Rqt2-4 factors target these rotated ribosomes with hybrid tRNAs specifically, allowing subsequent RQC reactions. We also demonstrated that yeast Rqt2 and its mammalian homolog ASCC3 plays a crucial role in RQC induced by the repeats of AAA codons, indicating that the crucial role of ribosome ubiquitination in RQC is conserved in eukaryotic cells.

## Results

### Rqt1-dependent ubiquitination triggers RQC pathway

**Fig. 1:**
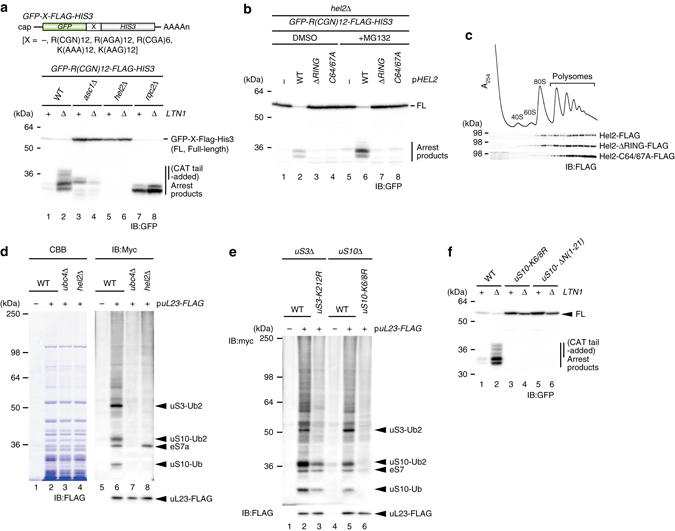
Hel2-dependent ubiquitination was required for a triggering step of RQC pathway. **a** An E3 ubiquitin ligase Hel2 was required for not only translation arrest, but also the production of arrest products. **b** The ubiquitination activity of Rqt1 was crucial for induction of RQC. **c** The distribution of Rqt1 proteins in polysome profiles. **d** The ubiquitinated proteins in ribosome depended on Hel2/Rqt1 and Ubc4. *Arrowed* proteins were identified by mass spectrometry. **e** K212 residue of uS3 and K6 and K8 residues of uS10 were ubiquitination sites of them. **f** Hel2-dependent ubiquitination of uS10-K6/8 was crucial for induction of RQC pathway

We have used well-established reporter system bearing repeated arginine codons R(CGN)12 to monitor the arrest products, which is substrate for RQC system (Fig. 1a and Supplementary Fig. 1a)^35^. An E3 ubiquitin ligase Ltn1 ubiquitinates the arrest products of R(CGN)12 reporter encoding poly-arginine sequence on the 60S subunit, and subsequently degraded by the proteasome; it was only detected in *ltn1*∆ mutant cells^16, 17, 35^ (Fig. 1a). Rqc2-dependent CAT-tails were also observed in *ltn1*∆ mutant cells (Fig. 1a). Mass spectrometry (MS) analysis revealed that peptides representing the arrested products ended with three or fewer C-terminal arginine residues (Supplementary Fig. 1b), and the CAT-tail was added mainly after two or three arginine residues in *ltn1*∆ mutant cells (Supplementary Data 1 and Supplementary Fig. 1b, e). The E3 ubiquitin ligase Hel2 has been identified to be involved in translation arrest induced by a poly-arginine sequence in genetic screens^2, 16^ (Supplementary Fig. 2a, b). R(CGN)12 reporter assay showed that full-length products indeed were significantly increased in *hel2* deletion mutant, and more interestingly, arrest products were completely disappeared in the *hel2*∆*ltn1*∆ double mutant (Fig. 1a), indicating that the ribosome could read through the R12 arrest sequence in the *hel2*∆ mutant owing to defective induction of RQC, so that it was not detected even in the absence of Ltn1; that is, as Hel2 apparently sorted the stalled ribosome from normal translation to RQC pathway, here we referred this protein as “RQC-trigger factor 1” (Rqt1).

Next, we assessed the role of ubiquitination by Rqt1 on the triggering step of RQC pathway. Its interacting E2 enzyme Ubc4 was required for the production of the arrested product in *ltn1*∆ mutant cells (Supplementary Fig. 2c, d), and Hel2C64/67A and Hel2∆RING mutant, which do not bind Ubc4, were also defective in the production of RQC substrates (Fig. 1b and Supplementary Fig. 2e), indicating that ubiquitination by Rqt1 is critical to induce the RQC pathway. In polyribosome profiles, Rqt1 as well as mutants defective in the ubiquitination were distributed in the heavy polysome fractions (Fig. 1c), implying that Rqt1 ubiquitinates unknown targets while bound to the 80S ribosome. To identify the targets of Rqt1, we purified ribosomes from myc-ubiquitin-expressing cells, and found three major bands that were ubiquitinated in an Rqt1-dependent manner (Fig. 1d). MS analysis revealed that these three bands were di-ubiquitinated uS3 and mono- or di-ubiquitinated uS10 at K212 of uS3 and K8 of uS10, respectively (Supplementary Fig. 3a, b). Substitution of the K212 residue of uS3 with arginine (K212R) disrupted its ubiquitination (Fig. 1e), and dual substitution of the K6 and K8 residues of uS10 with arginine (K6/8R) completely eliminated the ubiquitination of uS10 (Fig. 1e). Indeed, we reconstituted the ubiqutinations of uS10 by recombinant Rqt1 (E3), Ubc4 (E2), UBE1 (E1) enzymes, ubiquitin, and uS10-HA-tagged ribosomes in vitro (Supplementary Fig. 3c, d).

To assess the ubiquitination of uS3 and uS10 on the RQC pathway, we performed R(CGN)12 reporter assay. It clearly showed that the ribosome translated through the R12 arrest sequence, and the induction of RQC pathway was completely abolished like the *hel2*∆ mutant (Fig. 1f and Supplementary Fig. 3e, f), indicating that the ubiquitinations of uS10 at K6 and K8 are essential for the initiation of RQC system depended on Rqt1. This is consistent with recent studies by Hegde’s and Bennett’s lab have demonstrated that ZNF598, a mammalian homolog of Rqt1, ubiquitinates ribosome protein eS10, uS10, and uS3^33, 34^. Although we observed that the ubiquitination of uS3 was dependent of that of uS10 (Fig. 1e), the uS3 ubiqutination did not directly cause RQC (Supplementary Fig. 3e, g).

Next, we examined the linkage type of the ubiquitinations of uS10 and uS3. The overexpression of myc-Ubi-K48R led to the reduction of the uS10 di-ubiquitination level (Supplementary Fig. 4a), and the di-ubiquitinated uS10 was disappeared in uS10-K6/8 mutant by immunoblotting using K48 linkage-specific antibody (Supplementary Fig. 4b), indicating that the di-ubiquitination of uS10 occurs mainly through K48 ubiquitin linkages. Since these ubiquitinated proteins were not increased by MG132 treatment, it does not represent a signal for proteasomal degradation of the 40S subunit (Supplementary Fig. 4c). Taken together, our results indicated that Rqt1 recognizes stalled ribosomes and leads to the ubiquitination of uS10 for the induction of RQC, and the crucial role of ribosome ubiquitination in RQC is conserved in yeast and mammalian cells.

### RQC requires three ribosome quality control trigger factors

**Fig. 2:**
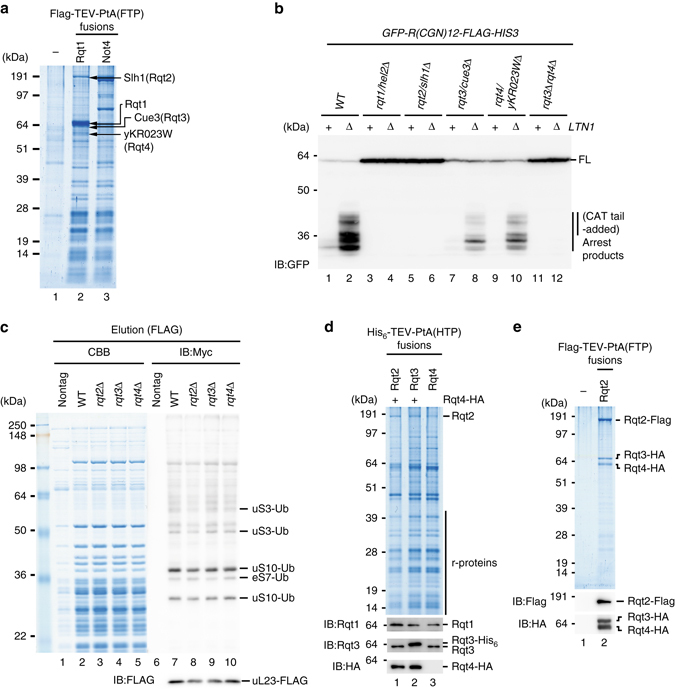
RQC requires three novel ribosome-quality control trigger factors (RQT). **a** Identification of RQT factors. Purified Rqt1-ribosome complex were analyzed by SDS-PAGE. *Arrowed* proteins were identified by mass spectrometry. **b** RQT factors played a crucial role in triggering step of RQC pathway. **c** Rqt2, Rqt3, and Rqt4 were dispensable for the ubiquitination of ribosome proteins by Rqt1. **d** Affinity purification of Rqt2-, Rqt3-, and Rqt4-ribosome complexes. **e** Rqt2, Rqt3, and Rqt4 formed a complex independent of ribosomes

To identify the putative factors involved in the subsequent steps of RQC, we purified Rqt1-ribosome complexes from cells expressing C-terminal *FLAG-TEV-ProtA*-tagged Rqt1 (Fig. 2a). Ubiquitinated uS10 species was specifically detected in ribosomes co-purified with Rqt1, suggesting that the complex is subjected to RQC (Supplementary Data 2). The most specific abundant proteins co-purified with the Rqt1-ribosome complex were Slh1 (Ski2-like helicase 1), Cue3 (coupling of ubiquitin conjugation to ER degradation 3), and the so far uncharacterized YKR023W (Fig. 2a) as identified by MS analysis. All three of these factors are required genetically for the efficient production of RQC substrates in vivo (Fig. 2b). This is consistent with recent study by Brandman’s lab^36^. Despite of weaker phenotype of a single deletion mutant of Cue3 and yKR023W, RQC substrates were not accumulated anymore in the double mutant of them (Fig. 2b), indicating their crucial function in the RQC-triggering step. Thus, we renamed them as Rqt2 (Slh1), Rqt3 (Cue3), and Rqt4 (YKR023W).

Although their phenotypes were very similar to Rqt1, these three proteins were not involved in uS10 ubiquitination (Fig. 2c), suggesting that they act downstream of Rqt1. All three factors were co-purified approximately equal amounts by affinity-purification via each of the individual proteins (Fig. 2d), and it was independent of the ubiquitination of uS10 by Rqt1 (Supplementary Fig. 5a). We further found that Rqt2-3-4 subcomplex was resistant to nuclease treatment during purification step (Fig. 2e), indicating that they formed a stable complex independent of ribosomes and thus we refer as “RQT complex”. As is often the case with multi-protein complex, the expression levels of Rqt3 and Rqt4 were drastically decreased in the *rqt2*∆ mutant (Supplementary Fig. 5b). Although Rqt2 protein level was slightly decreased in the *rqt3*∆, *rqt4*∆, or *rqt3*∆*rqt4*∆ mutants (Supplementary Fig. 5c, e), the association with ribosomes was nearly abolished even in the single deletion mutant of them (Supplementary Fig. 5d, e). Notably, the mRNA levels of the *GFP-R(CGN)12-HIS3* reporter were also slightly decreased in *rqt1*, *rqt2*, *rqt3*, *rqt4*, or *rqt3rqt4* deletion mutants (Supplementary Fig. 5f, g), but this reduction may not be able to account for the phenotype of these mutants in RQC. Collectively, Rqt2 is definitely essential for the RQC pathway, and Rqt3 and Rqt4 act as co-factors to form the fully functional RQT complex.

**Fig. 3:**
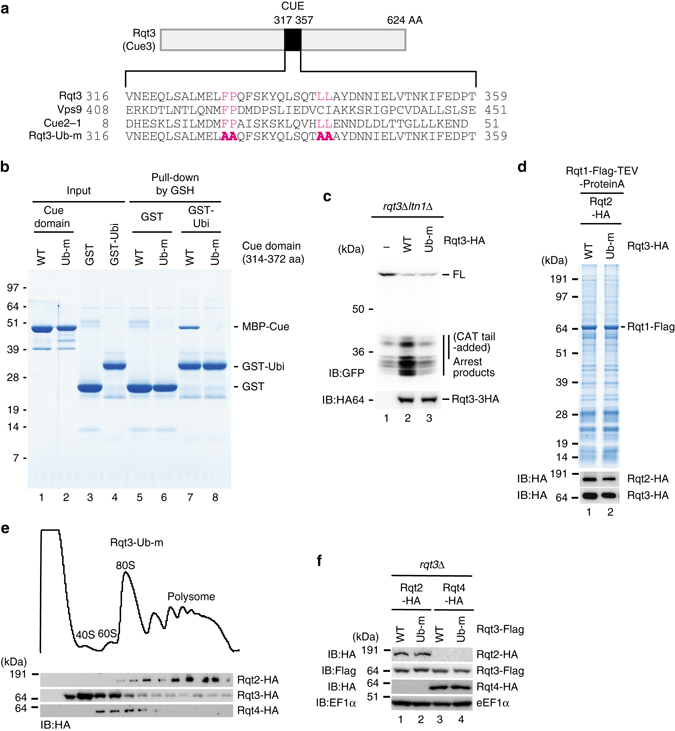
The mutations of Rqt3 in the residues crucial for the ubiquitin-binding domain diminished RQC. **a** Alignment of the conserved residues in the CUE domains of Rqt3, Vps9, and Cue2. The mutated residues in Rqt3-Ub-m were shown in *red*. **b** CUE domain of Rqt3 interacted directly with ubiquitin, and it was abolished by Ub-m mutations. **c** The mutations of Rqt3 in the residues crucial for the ubiquitin-binding domain diminished RQC. **d** The mutation in the ubiquitin binding of Rqt3 is dispensable for the association of Rqt2-4 in Rqt1-ribosome complex. **e** The distribution of Rqt2-4 proteins in polysome profiles of Rqt3-Ub-m mutant. **f** The expression levels of endogenously HA-tagged Rqt2 and Rqt4, Flag-tagged Rqt3 and Rqt3-Ub-m proteins in Rqt3-Ub-m mutant cells

Interestingly, Rqt3 has a CUE domain that binds to the ubiquitin (Fig. 3a), raising the possibility that the RQT complex could recognize the uS10 ubiquitination via Rqt3 to proceed to the RQC pathway. To confirm this possibility, we analyzed the direct interaction between CUE domain of Rqt3 and ubiquitin by in vitro-binding assay. As expected, CUE domain of Rqt3 was directly bound to ubiquitin, and it was completely abolished by the mutations (Ub-m) in the conserved residues of CUE domain (Figs. 3a, b). In addition, R(CGN)12 reporter assay revealed that this mutation significantly diminished the production of the RQC substrate (Fig. 3c). We further found that the RQT complex was recruited to the ribosome independent of Rqt3 ubiquitin-binding activity (Fig. 3d, e), and the expression of the RQT factors in Rqt3-Ub-m mutant cells was not reduced (Fig. 3f), implying that the ubiquitination of the stalled ribosome could be recognized by Rqt3 after recruitment of RQT complex.

**Fig. 4:**
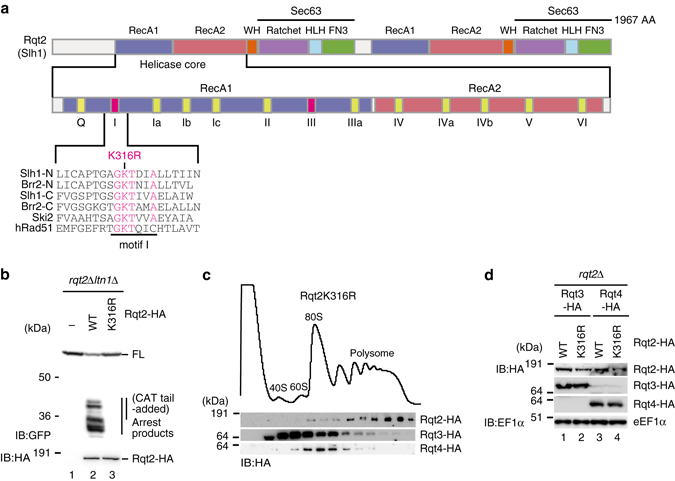
The mutations of Rqt2 in the conserved residues in ATPase domain diminished RQC. **a** Alignment of the conserved residues in the RecA1 motif I of Slh1/Rqt2, Brr2, Ski2, and hRad51. The substitution of the lysine to arginine residue of motif I in human Rad51 diminished the ATPase activity^37^. **b** The mutations of Rqt2 in the conserved residues in ATPase domain diminished RQC. **c** Rqt2-3 were distributed in heavy polysome fraction in Rqt2K316R mutant. **d** The expression levels of endogenously HA-tagged Rqt2, Rqt2-K316R, Rqt3, and Rqt4 proteins in Rqt2-K316R mutant cells

Rqt2 belongs to a family of ATP-dependent Ski2-like RNA helicases and contains two RecA-like helicase cassettes (Fig. 4a). We mutated a conserved lysine in the first helicase cassette to arginine (Rqt2-K316R), which leads to deficiency of ATP hydrolysis in the RecA family^37^, and analyzed its RQC activity. In this mutant, the production of the RQC substrate was completely eliminated like the *rqt2*∆ mutant (Fig. 4b), while ribosome-binding activity was still retained (Fig. 4c). The expression level and recruitment of RQT complex into ribosome were not affected by this mutation (Fig. 4c, d). These results suggest that the ATP-consuming reaction by the RQT complex occurs as a triggering step for the RQC pathway before ribosome dissociation.

### Stalled ribosomes in a rotated state are targets for RQC

**Fig. 5:**
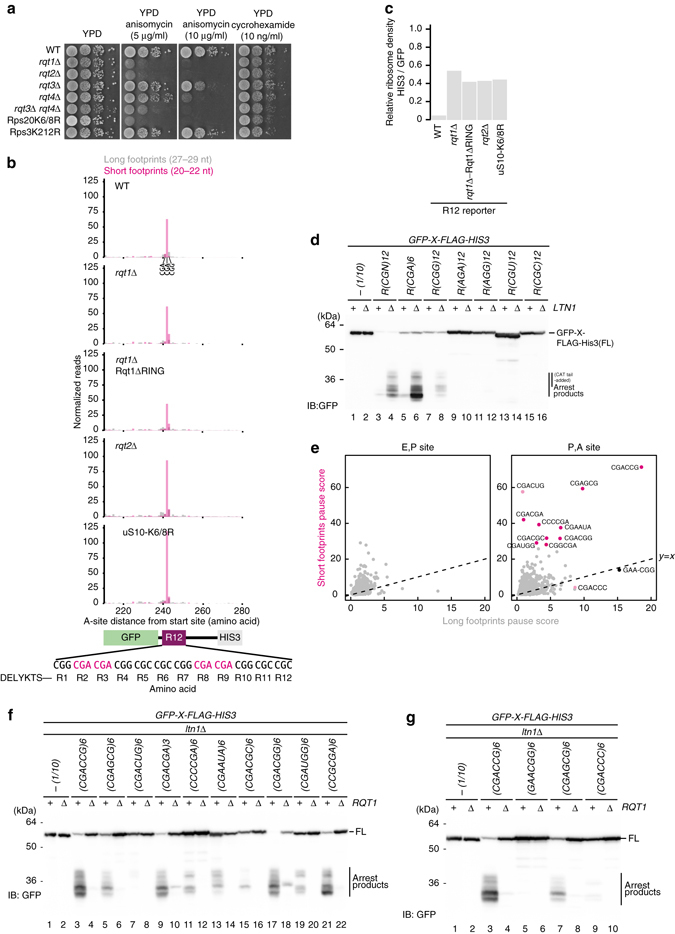
Stalled ribosomes in a rotated state conformation are targets for RQC. **a** The anisomycin sensitivity of *rqt* and uS10-K6/8R mutants. **b** Ribosome short footprints were accumulated at CGA-CGA codons. The short footprints and long footprints from the ribosome profiling data set were mapped on the reporter gene (*GFP-R12-FLAG-HIS3*). The reads were plotted at approximate position of ribosome A-site. **c** Translation of *HIS3* downstream of R(CGN)12 arrest-sequence was increased in the *rqt* and *uS10-K6/8R* mutants. **d** Codon specificity of poly-arginine sequence to induce translation arrest and RQC. **e** Specificity of di-codons for accumulation of short and long footprints. **f** *GFP-X-FLAG-HIS3* reporter assay of Top 10 di-codons in the accumulation of short footprints. **g** *GFP-X-FLAG-HIS3* reporter assay of top 4 di-codons in the accumulation of long footprints

It is largely unknown what characteristic of ribosomes the surveillance system recognizes to initiate RQC. The yeast *rqt1*∆ and *rqt2*∆ mutant cells conferred sensitivity to deoxynivalenol, trichothecin^38^, and anisomycin^16^ (Fig. 5a), which are drugs that bind to the peptidyl transferase center of ribosome and inhibit protein synthesis^39^. The correlation between the anisomycin sensitivities (Fig. 5a) and the defects in targeting stalled ribosomes to RQC (Fig. 2b) suggested that RQC plays a crucial role in rescuing the ribosome stalled by anisomycin and may provide a clue as to what conformational state of the ribosome is recognized by the RQT system.

An earlier study by ribosome profiling, which provides genome-wide analysis of translation with codon resolution^40^, has shown that two distinct populations of ribosome footprints with 20–22 nucleotide (nt) (short footprints) and 27–29 nt (long footprints), may represent the two different conformations of ribosomes in rotated and classical state, respectively^41^ (Supplementary Fig. 6a). Given the correspondence between the specific accumulation of short footprints by anisomycin^41^ and the sensitivity to the drug in *rqt* mutants (Fig. 5a), we reasoned that Rqt machinery targets the ribosome stalled at rotated state. Indeed, ribosome profiling data from yeast cells expressing the R(CGN)12 reporter also clearly showed the strong (~50-fold) accumulation of short footprints at CGA(R2)-CGA(R3) di-codons (Fig. 5b and Supplementary Fig. 6b). Consistently, we observed by MS analysis that peptides representing the arrested products ended with three or fewer C-terminal arginine residues (Supplementary Fig. 1b). Ribosomes seemed to stall specifically at CGA-CGA di-codons in the rotated state preventing the translating of the entire R(CGN)12 sequence (WT in Fig. 5b and Supplementary Fig. 6). Consistent with this, Grayhack and co-workers reported that CGA decoding by a single tRNA with inosine in the anticodon loop is crucial for the production of RQC substrate^42^. Consistent with western blot data as shown in Figs. 1 and 2, *rqt* deletion mutants and uS10 ubiquitination defective mutant showed recovered ribosome occupancy downstream of R(CGN)12 (Fig. 5c and Supplementary Fig. 6b, c). However, the mutants did not reduce the number of ribosomes stalled at CGA-CGA di-codons, and even caused more to accumulate in *rqt2* deletion mutant and uS10-K6/8R mutant (Fig. 5b and Supplementary Fig. 6c), and we expect that translation is stalled by R(CGN)12 sequence in the these mutants cells without the subunit dissociation, which then is needed to restart elongation.

Next, we asked whether polybasic amino acids in general or rather specific codon combinations are causing ribosome stalls, which lead the RQT-triggered RQC response. Metagene analysis of ribosome footprints around polybasic tracts from endogenous mRNAs showed that ribosomes paused in the non-rotated state ~8 amino acids downstream of the start of polybasic tracts, suggesting that the nascent polybasic tract can reach the exit tunnel for interaction (Supplementary Fig. 6d). However, we did not observe the accumulation of rotated state ribosomes around these polybasic tracts (Supplementary Fig. 6e). Poly-arginine reporter constructs coded by R(AGA)12, R(AGG)12, R(CGU)12, and R(CGC)12 did not accumulate arrested products in an *ltn1*∆ background, whereas the R(CGG)12 reporter moderately accumulated the arrested products in a Rqt1-dependent manner (Fig. 5d and Supplementary Fig. 7a). These data suggest that stalling by poly-arginine does not generally trigger RQC via the RQT system, and putative targets for RQT are rather ribosomes decoding specific combinations of codons. Thus, to examine whether top 10 di-codons in the accumulation of short footprints induce RQC or not, we constructed the reporter genes containing 6 repeats of 10 di-codons between *GFP* and *HIS3* genes, and detected the arrested products in an *ltn1*∆ background (Fig. 5e). All top 10 di-codons induced the production of the arrest products in *ltn1*∆ mutant cells in an Rqt1-dependent manner (Fig. 5f). We also examined whether top four di-codons in the accumulation of long footprints induce RQC. CGACCG and CGAGCG accumulated short and long footprints and induced RQC (Fig. 5f, g). However, GAACGG and CGACCC di-codons were accumulated only long footprints, and induced no or low RQC (Fig. 5g). These data indicated that the accumulation of short footprints could be a feature of RQC targets, but not long footprints. Since 6 of the top 10 di-codons in the accumulation of short footprints are included in the recently discovered 17 translational inhibitory di-codons^43^, we also examined whether other 11 inhibitory di-codons induced RQC, and found that only 2 of them induced RQC (Supplementary Fig. 7b, c), suggesting that neither the accumulation of long footprints nor translation inhibition is sufficient to induce RQC. These findings lead us to propose that specific di-codons in P and A sites drastically facilitate the accumulation of stalled ribosomes in the rotated form, leading to the production of RQC substrate.

### Rqt1-bound ribosomes are the rotated form with hybrid tRNAs

**Fig. 6:**
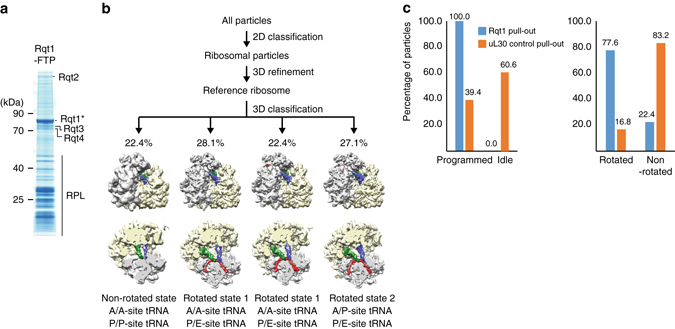
Rqt1-bound ribosomes are the rotated form with hybrid tRNAs. **a** CBB-stained SDS gel and Amidoblack-stained blot of pullouts from Rqt1-FTP strains. **b** Particle sorting was done by 3D classification in RELION using four classes starting from 142,001 particles. This resulted in three dominant classes with rotated ribosomes (two classes in rotated state 1 and one class in rotated state 2) and one class with un-rotated (classical) state ribosomes; the highest-resolution close-to-focus data set was refined using movie processing (see “Methods” section). In total, the data set contained 22.4% classical state and 77.6% rotated state ribosomes. **c** Population of programmed and unprogrammed ribosomes in Rqt1-FTP and uL30-TAP pullouts

In order to examine whether Rqt1 indeed binds rotated ribosomes, we purified native Rqt1-bound ribosomes by their association with Rqt1-FLAG-TEV-ProtA derived from the endogenous-tagged *RQT1* allele (Fig. 6a), and performed cryo-EM and single particle analysis. Unfortunately, we were not able to unambiguously identify Rqt1 in these complexes most likely due to flexibility. However, computational sorting showed that the vast majority of Rqt1-associated ribosomes contained programmed ribosomes occupied with two tRNA, indicating that most Hel2-asociated ribosomes are indeed translating ribosomes, and it is consistent with our initial finding of Rqt1 migrating deep in density gradients with the polysome fraction (Fig. 1c). Moreover, more than 77% of these ribosomes were found in a rotated state representing either the PRE state with hybrid A/P- and P/E-site tRNAs (rotated-2) or the rotated PRE state with A/A- and P/E-site tRNAs (rotated-1) (Fig. 6b, c and Supplementary Fig. 8). In contrast, a cryo-EM analysis of a control pullout via uL30 under the same conditions revealed majority of programmed ribosomes were in a non-rotated state (Fig. 6b, c and Supplementary Fig. 8). This indicates that Rqt1 indeed targets programmed ribosomes stalled during elongation in a defined ribosomal hybrid state.

### Rqt1 and Rqt2 are conserved in mammalian cells

**Fig. 7:**
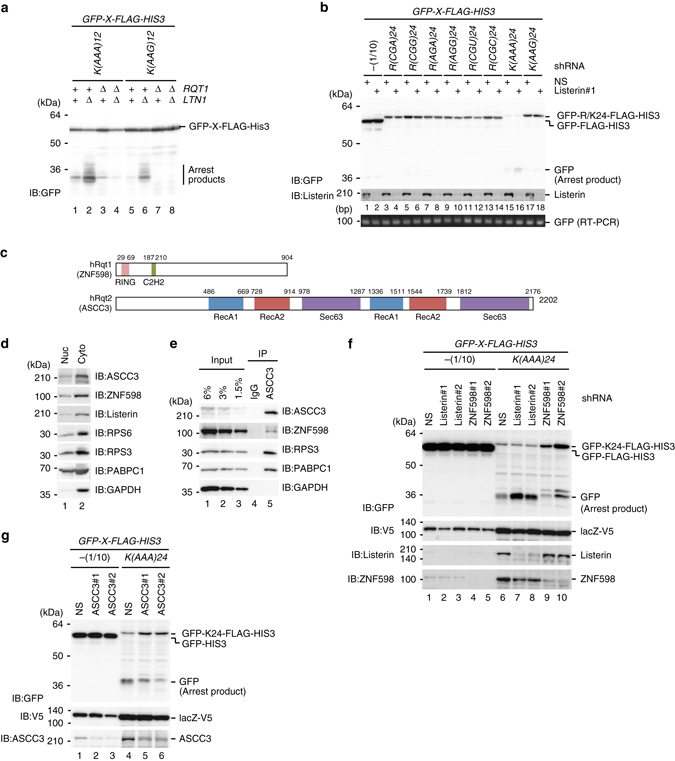
ASCC3, a human homolog of yeast Rqt2 is required for RQC induced by poly(A) sequence. **a** Rqt1 factors were required for RQC induced by poly-lysine sequences in yeast. **b** The AAA repeat induces RQC in mammalian cells. **c** Schematic drawing of alignment of the domain structure of ZNF598/hRqt1 and ASCC3/hRqt2. **d** ZNF598 and ASCC3 were localized mainly in cytosol. **e** ZNF598 was co-purified with ASCC3. **f** ZNF598 is involved in translation repression and the production of RQC substrate by K(AAA)24 sequence. **g** ASCC3 is a homolog of Rqt2 in mammalian cells

We demonstrated that specific di-codons, including CGA-CGA, in PA site drastically facilitate the accumulation of stalled ribosomes in the rotated form, leading to the production of RQC substrate. We next confirmed the functions of Rqt factors in RQC by the repeats of AAA were required for RQC in yeast (Fig. 7a). It has been reported that the AAA repeat induces translation arrest and RQC in mammalian cells^23, 33^, and we asked whether other poly-arginie or lysine sequences induced RQC or not. The reporter assay revealed that repeats of any arginine codons could not induce significant translation arrest, but K(AAA)24 strongly induced translation stalling (Fig. 7b). Since CGA codon in human cells is not decoded by wobble I-A base pair, CGA codon is not crucial for the production of RQC substrate (Fig. 7b).

We next examined the role of human homolog of Rtq factors in RQC by the AAA repeat. The zinc-finger protein 598 (ZNF598) is a component of the mammalian eIF4E homologous protein (m4EHP) complex that is proposed to be translation repressor by the competition with eIF4E for the cap structure^44^ (Fig. 7c). Recent study demonstrated that ZNF598 is a homolog of Rqt1 in mammalian cells, and involved in translation repression and the production of RQC substrate^33^. An activating signal co-integrator complex subunit 3 (ASCC3) is a DNA helicase and unwinding by ASCC3 is coupled with ALKBH3-dependent DNA alkylation repair and cancer cell proliferation in mammalian cells^45^. Since ASCC3 is highly homologous to yeast Rqt2, we examined the physical interaction between ASCC3 and ZNF598. Both of them were localized in cytosol (Fig. 7d), and it is consistent with their functions in RQC induced by aberrant translation. In addition, immunoprecipitation of ASCC3 showed that ASCC3 was indeed co-purified with ZNF598 and ribosomal protein uS3 (Fig. 7e), suggesting that the association of Rqt2 with Rqt1-bound ribosome is conserved in mammalian cells.

As the physical interaction between ZNF598 and ASCC3 was conserved in mammalian cells, we next examined the role of ASCC3 on RQC system. As expected, K(AAA)24 reporter assay showed that Listerin (Ltn1 homolog) knockdown led to accumulation of arrest products (Fig. 7f), in contrast, the reduction of arrest products were observed in ZNF598 or ASCC3 knockdown cells concomitant with an increase of full-length products (Fig. 7f, g). This is consistent with yeast data set, concluding that ASCC3 is a homolog of Rqt2 in mammalian cells, and involved in translation repression and the production of RQC substrate. These strongly suggest the conserved function of ASCC3 in RQC induced by the repeat of AAA codons in mammalian cells.

## Discussion

Aberrant ribosome stalling induces quality controls to prevent the production of potentially harmful products; however, the mechanisms and factors responsible for the recognition and dissociation of stalled ribosomes into subunits remain to be elucidated. Asc1 and Hel2 are involved in translation arrest^2, 16, 30^. K63-linked ubiquitination by Hel2 is proposed to be crucial for translation arrest^16^, however, its role in RQC and its substrates remains to be determined in yeast. Recent study demonstrated that the ubiquitination of ribosome plays a crucial role in translation arrest by poly(A) sequence K(AAA)24 in mammalian cells^33, 34^. The E3 ubiquitin ligase ZNF598 is required for translation arrest by K(AAA)24 sequence, and the ubiquitinations of eS10 at K138/139 or uS10 at K4/8 by ZNF598 is involved in translation arrest by poly-lysine sequence^33, 34^. However, the substitutions of ribosome protein eS10 or uS10 ubiquitination sites impaired translation arrest partially^33, 34^, indicating that translation arrest by poly(A) sequence depends on ribosome ubiquitination at multiple sites by ZNF598 in mammalian cells. In this study, we identified the ubiquitination sites of ribosome by Hel2 and demonstrated its essential role in the production of Ltn1 substrate induced by various translation arrests (Figs. 1, 5, and 7 and Supplementary Fig. 7), indicating that ZNF598 and its yeast homolog Hel2/Rqt1 ubiquitinates ribosome protein to induce RQC. The mono- or di-ubiquitination of uS10 lysine residues was essential for RQC (Fig. 1f), and the ubiquitinated ribosomes were not targeted for the degradation (Supplementary Fig. 4c). On the basis of these results, we propose that the modification at the adjacent sites of ribosome protein uS10 is required for triggering steps including the subunit dissociation in RQC in yeast. The Hel2-dependent ubiquitination of uS3 at K212 was largely diminished in the uS10-K6/8R mutant cells, but the substitution of lysine 212 of uS3 to arginine did not affect the ubiquitination of uS10 and RQC (Fig. 1e and Supplementaly Fig. 3f, g). The role of uS3 ubiquitination in other quality controls or translation regulation remains to be elucidated.

An important and unsolved question is how the ubiquitinated ribosome is rerouted to the subunit dissociation for Ltn1-dependent ubiquitination of peptidyl-tRNA on 60S subunit. We identified the novel RQT complex that associates with ribosome and plays a crucial role in RQC (Figs. 2–4). The RQT complex is not required for the ubiquitination of uS10 at K6 and/or K8 residues (Fig. 2c), indicating that the RQT complex acts downstream of the Hel2-dependent ubiquitination of uS10. In addition, the association of Rqt complex with Hel2/Rqt1-bound ribosome is independent of the ubiquitination of uS10 by Hel2/Rqt1 (Supplementary Fig. 5a), and three Rqt proteins form a complex independent of ribosome (Fig. 2e). These suggest that the ribosome ubiquitination is not pre-request for the association of Rqt complex to the ribosome. Importantly, the ubiquitin-binding activity of Rqt3 is crucial for RQC (Fig. 3), suggesting that Rqt3 is responsible for the recognition of the ubiquitinated uS10 at K6 and/or K8 residues to initiate RQC. The ATPase activity of Rqt2 is also required for RQC (Fig. 4), indicating that Rqt2 plays an essential role in RQC with utilizing energy of ATP hydrolysis. Rli1, ABCE1 in mammal, interacts with eRF1 and facilitates the subunit dissociation with utilizing its ATPase activity in normal translation cycle^3, 7, 46, 47^. Rli1 also interacts with Dom34 in yeast, ABCE1 interacts with Pelota in mammal, to induce the dissociation of ribosome stalling at the 3′ end of aberrant nonstop mRNA thereby playing a crucial role in NSD and RQC^3, 6–9, 46, 47^. One possibility is that Rqt2 is directly involved in the subunit dissociation with utilizing its ATPase activity, and the interaction of Rqt3 with the ubiquitinated ribosome at uS10 is a triggering step to activate ATPase activity of Rqt2 to dissociate the stalled ribosome. Interestingly, Ski2 and Slh1p block translation of mRNA lacking a poly(A) tail by an effect on Fun12^48, 49^, suggesting that the involvement of Rqt2 in quality control or translation of aberrant mRNA. Biochemical and structural approaches are required to uncover the precise functions of these novel factors involved in RQC.

Emerging evidences indicate that RQC depends on the codon-specific translation arrest. In yeast, ribosome stalling at six repeats of CGA arginine rare codons results in Ltn1-dependent degradation of the arrest product, but the repeat of optimal arginine AGA codons represses translation but the arrest product was hardly detected even in the *ltn1* mutant^14, 42, 50, 51^. CGA codon is decoded by tRNA^Arg(ICG)^ with A-I wobble interaction, suggesting that translation repression by poly-arginine sequence is not sufficient but the specific codons are responsible for RQC. A comprehensive analysis of the inhibitory effects of di-codons in yeast identified 17 di-codon pairs linked to translation arrest, and elevated the levels of long footprints (27–29 nt)^43^. These findings implicate interplay between P sites and A sites in codon pair-mediated inhibition.

In this study, we demonstrated that CGA-CGA di-codon at P-A site drastically accumulated short footprints (20–22 nt) in R12 sequence (Fig. 5b). We have examined whether RQC is correlated with the levels of short footprints accumulated at di-codons, and found that all top 10 di-codons that accumulated short footprints induced RQC (Fig. 5f, g). In contrast, GAACGG di-codon that accumulated only long footprints did not induce RQC (Fig. 5g). Seventeen di-codon pairs linked to translation arrest, and elevated the levels of long footprints but only eight of them induced RQC (Supplementary Fig. 7b, c), indicating that the specific di-codons to induce translation inhibition is not essential for RQC. These results clearly demonstrated that the accumulation of short footprints is the feature of RQC targets, but not long footprints, which is the feature of general ribosome stalling. Consistently, cryo-EM analysis revealed that Hel2-bound ribosome is the rotated form with hybrid tRNAs (Fig. 6). We propose that the specific di-codons in PA site drastically facilitate the accumulation of stalled ribosome specifically in the rotated form, and Rqt1 primarily recognizes the rotated ribosome and ubiquitinated uS10 at K6/8 residues, leading to Rqt complex-dependent production of Ltn1 substrate (Supplementary Fig. 9).

The poly-lysine or -arginine sequences strongly repress translation, and the efficiency of repression varies depending on the codons, and poly(AAA) triggers translation arrest much more robustly than poly(AAG)^13, 23, 30, 35^. More than 8 lysine codons are required to repress translation efficiently, and 12 lysine codons repress translation 20-fold and induce RQC efficiently^13^. There is no more than eight consecutive lysine codons in coding sequences that strongly accumulated long or short footprints (Supplementary Fig. 6e), suggesting that there is no endogenous poly(A) sequences that induce strong ribosome stalling, and a poly(A) tail of nonstop mRNA is a major endogenous poly-lysine sequence to induce translation repression and quality-control mechanisms. The ubiquitination of uS10 by Hel2/Rqt1 and Rqt complex are essential for RQC by K(AAA)12 sequence in yeast (Fig. 7a), and it is consistent with the role of eS10 ubiquitination by ZNF598 in mammalian cells^33^. The arrest products were detected in the Listerin knockdown cells, indicating ribosome stalling by K(AAA)24 induces RQC (Fig. 7f). We demonstrated that ASCC3 is a mammalian homolog of Rqt2, and required for the production of substrate of Listerin ligase and translation arrest by K(AAA)24 sequence (Fig. 7g), indicating that role of ribosome ubiqutination in RQC is conserved in eukaryotic cells (Supplementary Fig. 9).

The sensitivity of the *rqt* mutants to anisomycin correlated with the defects in RQC (Figs. 2b and 5a), suggesting that Rqt1 might recognize the rotated ribosome that is stalled by anisomycin and ubiqutinates uS10, leading to the production of RQC substrate by Rqt complex. Other drug or stress conditions may increase stalling ribosomes that are potential substrates for RQT factors, and the rescue of stalling ribosome may play crucial roles in the proper response to stresses. Indeed, the unfolded protein response (UPR) stimulation induces site-specific ubiquitination of ribosomal proteins including uS10 and uS3^52^. Further studies will be warranted to elucidate the cellular and molecular consequences of ribosome ubiquitination in UPR and other stress responses.

## Methods

### Yeast strains and genetic methods and plasmid construct

The *S. cerevisiae* strains and plasmids used in this study are listed in Supplementary Data 3, and were obtained by established recombination techniques^53, 54^.

### Western blot

Proteins in samples were separated by SDS-PAGE or Nu-PAGE, and were transferred to PVDF membranes (Millipore; IPVH00010). After blocking with 5% skim milk, the blots were incubated with the primary antibodies listed in the Supplementary Data 3. The secondary antibodies used in this study were conjugated with horseradish peroxidase, and detected by ImageQuant LAS4000 (GE Healthcare). Uncropped western blots are shown in Supplementary Fig. 10.

### Polysome analysis

Yeast cells were grown exponentially at 30 °C and treated 0.1 mg/ml of cycloheximide for 5 min before harvesting, then harvested by centrifugation. The harvested cell pellet was frozen and ground in liquid nitrogen using a mortal. The cell powder was resuspended with lysis buffer (20 mM HEPES-KOH, pH 7.4, 100 mM potassium acetate, 2 mM magnesium acetate) to prepare the crude extracts. Sucrose gradients (10–50% sucrose in 10 mM Tris-acetate, pH 7.4, 70 mM ammonium acetate, and 4 mM magnesium acetate) were prepared in 25 × 89 mm polyallomer tubes (Beckman Coulter) using a Gradient Master. Crude extracts (the equivalent of 50 A_260_ units) were layered on top of the sucrose gradients and then centrifuged at 150,000×*g* in a P28S rotor (Hitachi Koki, Japan) for 2.5 h at 4 °C. The gradients were then fractionated (TOWA Lab, Tsukuba). The polysome profiles were generated by continuous absorbance measurement at 254 nm using a single path UV-1 optical unit (ATTO Biomini UV-monitor) connected to a chart recorder (ATTO digital mini-recorder). Equal volumes of the fractions were collected and processed for western blotting.

### Ribosome purification to identify the Rqt1 targets

To identify the putative target(s) of Rqt1, we co-expressed Myc-tagged ubiquitin (Myc-Ubi), which is partially resistant to proteasomal degradation^55^, and the FLAG-tagged ribosomal protein uL23 (uL23-FLAG). Yeast cells harboring p*CUP1*p-*MYC-UBI* and p*RPS2(uS5)-FLAG* or p*RPL25(uL23)-FLAG* were cultured in 800 ml of synthetic complete medium. To induce the expression of Myc-Ubi, the cells were cultured in the presence of 0.1 mM Cu^2+^ for 2 h. Cell lysates were prepared and FLAG-tagged ribosomes were purified using M2 FLAG-affinity resin (Sigma), as described previously^56^. Affinity purified samples were subjected to SDS-PAGE followed by western blotting with an anti-Myc antibody. The sections of the gels corresponding to the bands detected by western blotting were isolated and analyzed by mass spectrometry^57^.

### Identification of Rqt1 targets and ubiquitination sites

After the gels were extensively washed with Milli-Q water (Millipore), the gel band was excised, diced into 1 mm^3^ pieces, and destained by 1 ml of 50 mM ammonium bicarbonate (AMBC)/30% acetonitrile (ACN) with agitation for 1 h, then the gels were further washed by 1 ml of 50 mM AMBC/50% ACN for 1 h. Finally, 100% ACN wash was performed to ensure complete gel dehydration. Trypsin solution (Promega, 20 ng/µl in 50 mM AMBC) was subsequently added to gel pieces at approximately equivalent volume and incubated on ice for 30 min. Another small volume of trypsin solution was added to gel samples and incubated at 37 °C for overnight. Digests were extracted by addition of 50 μl of 50% ACN/0.1% trifluoroacetic acid (TFA) for 1 h by shaking. The digested peptides were recovered into fresh eppendorf tubes and additional extraction step was performed with 70% ACN/0.1% TFA for 30 min. The extracted peptides were combined and concentrated by a speed-vac. After centrifugation, the recovered peptides were analyzed by nanoflow HPLC (Easy nLC1000, Thermo Scientific) coupled with a Q Exactive mass spectrometer (Thermo Scientific). The mobile phases were 0.1% formic acid (FA) in water (solvent A) and 0.1% FA in 100% ACN (solvent B). Peptides were directly loaded onto a C18 analytical column (ReproSil-Pur 3 μm, 75 μm inner diameter, and 12 cm length, Nikkyo Technos) and separated using a 80 min two step gradient (0–35% in 70 min, 35–100% in 10 min, and 100% in 10 min of solvent B) at a constant flow rate of 300 nl/min. The Q Exactive was operated in the data-dependent MS/MS mode, using Xcalibur software, with survey scans acquired at a resolution of 140,000 at *m/z* 200. The top 10 most abundant isotope patterns with charge 2–5 were selected from the survey scans with an isolation window of 2.0 *m/z*, and fragmented by HCD with normalized collision energies of 28. The maximum ion injection times were 60 ms for both survey and MS/MS scans, and the AGC values were set to 3 × 10^6^ and 5 × 10^5^ for the survey and MS/MS scans, respectively. Ions selected for MS/MS were dynamically excluded for 10 s.

Proteome Discoverer software (ver. 1.3, Thermo Scientific) was used to generate peak lists. The MS/MS spectra were searched against a SwissProt database (version 2012_10 of UniProtKB/Swiss-Prot protein database) using the MASCOT search engine. The precursor and fragment mass tolerances were set to 10 ppm and 20 mmu, respectively. Methionine oxidation, protein amino-terminal acetylation, pyroglutamate formation, serine/threonine/tyrosine phosphorylation, and diglycine modification of lysine side chains were set as variable modifications, and cysteine methylthio modification was set as a static modification for database searching. Peptide identification was filtered at a 1% false discovery rate. We also analyzed the MS data by using Paragon search engine. The raw files were converted to MGF files using PD1.3 and were searched by ProteinPilot software (ver. 4.0, AB Sciex) against the SwissProt database.

### Purification of Rqt factors associated ribosome

The yeast strains expressing the C-terminal protein A fused each *RQT* gene, which are *RQT1-FTP* (Flag-TEV-ProteinA), *RQT2-HTP* (His_6_-TEV-ProteinA), *RQT3-HTP*, or *RQT4-HTP*, were cultured in 4L YPD medium. The harvested cell pellet was frozen in liquid nitrogen, and then ground in liquid nitrogen using a mortal. The cell powder was resuspended with lysis buffer (50 mM Tris, pH 7.5, 100 mM NaCl, 10 mM MgCl_2_, 0.01 % NP-40, 1 mM DTT) to prepare the lysate. Purifications were performed as modified version of described before^58^. The lysate was centrifuged at 39,000×*g* for 30 min at 4 °C, and the supernatant fraction was used for the purification step. The Rqt factors associated ribosomes were affinity purified using IgG-conjugated Dynabeads (Invitrogen), followed by TEV protease cleavage to release them. The TEV elution was incubated with Ni-NTA beads (Novagen) to remove the TEV-protease.

### Purification of RQT complex

The yeast strain overexpressing the *RQT2-FTP*, *RQT3-3HA*, and *RQT4-3HA* were cultured in 4L synthetic complete medium. The harvested cell pellet was frozen in liquid nitrogen, and then ground in liquid nitrogen using a mortal. The cell powder was resuspended with lysis buffer (50 mM Tris pH 7.5, 100 mM NaCl, 5 mM MgCl_2_, 0.01 % NP-40, 10% glycerol, 1 mM DTT) to prepare the lysate. The lysate was centrifuged at 39,000×*g* for 30 min at 4 °C, and the supernatant fraction was used for the purification step. Purifications were performed as modified version of described before^58^. The RQT complex was affinity purified using IgG beads (GE Healthcare), followed by TEV protease cleavage to release it. The TEV elution was further incubated with benzonase (MERCK) to digest the ribosome, and then re-purified by FLAG-M2 affinity resin (Sigma).

### In vitro ubiquitin-binding assay of Rqt3 cue domain

The maltose binding protein (MBP)-fused cue domain of Rqt3 was expressed by using pETM40-Rqt3, and the GST-fused ubiquitin was expressed by using pGEX-Ub in *E. coli* Rosetta-gami 2 (DE3) cells. The each transformed cell was grown at 23 °C in LB medium until they reached an absorbance at 600 nm (A_600__nm_) of 0.6, isopropyl-b-D-thiogalactoside was added to a final concentration of 0.1 mM. The cells were grown for an additional 3 h and then collected by centrifugation and stored frozen at −80 °C. Frozen pellets were resuspended in buffer (25 mM Tris, pH 7.5, 100 mM NaCl, 1 mM MgCl_2_, 0.01% NP-40 and 2 mM β-mercaptoethanol) with protease-inhibitor cocktail (Roche), and were broken by sonication on ice. The lysate was centrifuged at 39,000×*g* for 30 min at 4 °C. The supernatant fractions were used for the purification step. The MBP-cue domain and GST-ubiquitin were purified by amylose resin (NEB) or GSH resin (GE Healthcare), respectively, and the both columns were washed with PBS buffer, including 1% Triton X-100 before elution. MBP-cue domain was eluted by the addition of 50 mM maltose and then mixed with immobilized GST or GST-ubiquitin GSH resin, followed by incubation for overnight at 4 °C. The column was washed with PBS buffer including 1% Triton X-100 and eluted by 20 mM glutathione. The final elution was analyzed by SDS-PAGE.

### Ribosome profiling and data analysis

Library preparation was performed according to the method previously described^59^ with following modifications. The whole cell lysate containing 20 μg of total RNA was treated with 10 units of RNase I (Epicentre) at 24 °C for 45 min. A linker DNA, 5′-(Phos)NNNNNIIIIITGATCGGAAGAGCACACGTCTGAA(ddC)-3′, where (Phos) indicated 5′ phosphoryaltion and (ddC) indicates a terminal 2′,3′-dideoxycytidine, was used. The Ns and Is indicate random barcode for eliminating PCR duplication and multiplexing barcode, respectively. The linkers were pre-adenylated with 5′ DNA Adenylation kit (NEB), and then used for the ligation reaction. Un-reacted linkers were digested by 5′ deadenylase (NEB) and RecJ exonuclease (epicentre) at 30 °C for 45 min. An oligo 5′-(Phos)NNAGATCGGAAGAGCGTCGTGTAGGGAAAGAG(iSp18)GTGACTGGA GTTCAGACGTGTGCTC-3′, where (Phos) indicated 5′ phosphorylation and Ns indicate random barcode, was used for reverse transcription. PCR was performed with oligos, 5′-AATGATACGGCGACCACCGAGATCTACACTCTTTCCCTACACGACGCTC-3′ and 5′-CAAGCAGAAGACGGCATACGAGATJJJJJJGTGACTGGAGTTCAGACGTGTG-3′, where Js indicate reverse complement of the index sequence discovered during Illumina sequencing.

The libraries were sequenced on a HiSeq 2000/4000 (Illumina). Reads were mapped to reporter sequence and yeast transcriptome, removing duplicated read based on random barcode sequences. The analyses were restricted to 20–22 and 27–29 nt long reads. We empirically estimated the position of A-site from 5′-end of the reads based on the length of each footprint. The offsets were 16 for 22 and 29 nt reads and 15 for 20, 21, and 28 nt reads, and 14 for 27 nt reads.

Ribosome pause scores of short and long footprints were calculated as previously published^60^. Basically, we took short or long footprints accumulation over the average of the footprint density in the given ORFs avoiding first and last five codons. Analyses were restricted to the mRNAs with 0.5 footprints/codon and more. The averaged pause scores of short and long footprints on given di-codons were computed. For metagene analysis, we defined polybasic tract as the amino acid sequence with six or more arginine or lysine in 10 amino acid window, as previously published^2^.

### Relative quantification of arrested peptides by MS

A R12 reporter, ProtA-TEV-GFP-R(CGN)12-Gas1, was expressed and isolated from WT or *ltn1*Δ cells. Soluble arrested proteins purified as above were precipitated by cold-acetone, resolubilized with 50 mM AMBC, and digested with AspN protease (Promega, 1:50 w/w) for 2 h at 37 °C. The reaction was terminated by acidification with 0.1% TFA, and the peptide cleavage was confirmed by SDS-PAGE. First, we performed the Top 10 analysis as described above using a 30 min two steps gradient (0–35% in 25 min, 35–100% in 5 min, and 100% in 8 min of Solvent B) at a constant flow rate of 300 nl/min. The raw data were analyzed by De Novo sequencing software PEAKS 8 (Bioinformatics Solutions Inc.) to identify arrest sites and CAT. Next, we performed quantitative analysis of arrested peptides using MS1 ions. The Q Exactive was operated in the full MS scan method with an *m/z* 300 to 1500 scan range, an orbitrap resolution of 140,000 at *m/z* 200, target automatic gain control values of 3 × 10^6^, and maximum fill times of 200 ms. Data analysis was performed using Pinpoint software (ver. 1.3, Thermo Scientific) with a data set of possible arrested sequences of R12 reporter, DELYKTSR*x* (0 < *x* < 12) and DELYKTSR*x* (A or T)*y* (*x* < 3, 0 < *x* + *y* < 12) (Supplementary Table 2). Mass tolerance was set at 10 ppm for extraction of ion chromatograms, and MS1 intensities, sum of doubly and triply charged masses of each peptide, were exported as a CSV file and further analyzed by Microsoft Excel.

### Cryo-EM analysis of Rqt1-ribosome complex and uL30 pullout

Yeast cell expressing Rqt1-FTP (Flag-TEV-Protein A) were cultured in YPD medium. As a control sample, a yeast strain containing genomically tandem-affinity purification (TAP) tagged uL30 was also expressed in YPD. Purifications were performed as described before^4^. The Rqt1 complex and uL30 control were purified from whole-cell lysates by affinity purification using IgG-conjugated dynabeads, followed by TEV protease cleavage to elute it. Freshly prepared sample was applied to 2 nm pre-coated R3/3 holey carbon-supported grids (Quantifoil) and vitrified using a Vitrobot Mark IV (FEI Company). Data were collected on a Titan Krios TEM (FEI Company) equipped with a Falcon II direct electron detector, operated at 300 keV. The magnification settings resulted in a pixel size of 1.084 Å/pixel. The data set was provided with the semi-automatic software EPU (FEI Company) with an accumulated dose of ~28 e^−^/Å^2^ divided on 10 frames. Frames were aligned using Motion Correction software^61^ and CTF estimation was performed with CTFFIND4. Particles were picked using Gautomatch (http://www.mrc-lmb.cam.ac.uk/kzhang/). Data processing was done with RELION. In brief, 2D classification was performed to exclude non-ribosomal particles. The remaining particles were subsequently refined and 3D classified to obtain classes with classical or rotated ribosomes. Images were made with UCSF chimera.

### Cell culture, lentiviral transduction, and transfection

HEK293T cells were cultured in DMEM medium supplemented with 10% fetal bovine serum. Gene knockdown experiments were performed using shRNA-expressing lentiviruses^62^. Targeting sequences of shRNA were as shown below, 5′-GGAGACGTATATGTTGAAA-3′ (shListerin-1), 5′-GGTGCTGCGGAAACTTTCAAA-3′ (shListerin-2), 5′-GCCAGTTGCCGTCGTCGTTAAT-3′ (shZNF598-1), 5′-GCCCAGGACTACTACAGCGACTAT-3′ (sh ZNF598-2), 5′-GATGCAGACGTTGAAAAAATA-3′ (shASCC3-1), 5′-GTAATGCTACTAATCGAATTA-3′ (shASCC3-2). Transfection of plasmid DNA listed in Supplemenraly Data 3 was carried out using PEI-max reagent according to the manufacturer’s instruction (Cosmo Bio).

### Nuclear and cytoplasmic fractionation

HEK293T cells (10 cm dish) were washed with ice-cold PBS, scraped from the dish, and collected by a brief centrifugation. The cells were lysed with 0.9 ml of buffer A containing 10 mM HEPES-KOH, pH 7.4, 15 mM KCl, 2 mM MgCl_2_, 0.1 mM EDTA, 0.1% IGEPAL-CA630, and 1 mM dithiothreitol supplemented with protease inhibitors (Roche) for 10 min on ice. The cytoplasmic extract was collected by centrifugation at 1000×*g* for 10 min at 4 °C. The pellet was resuspended in 0.3 ml of RIPA buffer containing 25 mM Tris-HCl, pH 7.5, 150 mM NaCl, 1% IGEPAL-CA630, 1% sodium deoxycholate, 0.1% SDS supplemented with protease inhibitors, incubated for 30 min on ice, and centrifuged at 18,000×*g* for 20 min at 4 °C. The supernatant was collected as nuclear extract.

### Immunoprecipitation

Immunoprecipitation was performed as described previously^62^. Briefly, HEK293T cells (10 cm dish) were homogenized with IP buffer containing 25 mM HEPES-KOH, pH 7.4, 150 mM NaCl, 5 mM MgCl_2_, 1 mM EDTA, and 1% IGEPAL-CA630 supplemented with protease inhibitors. The homogenates were incubated on ice for 30 min and centrifuged at 15,000×*g* for 10 min at 4 °C. The total cell lysates were mixed with 1 μg of antibody and rotated at 4 °C for 1 h. Protein G dynabeads (Invitrogen) was added to the mixture and further incubated at 4 °C for 12 h. After the beads were washed with IP buffer four times, bound proteins were analyzed by western blot using antibodies as listed in Supplementary Data 3.

### Data availability

The sequencing data for ribosome profiling experiments have been deposited in NCBI’s Gene Expression Omnibus and is accessible through GEO series accession number GSE79622, GSE90919, and GSE90920. Cryo-EM maps of the Rqt1-FTP pullout data set 3D classification scheme in Fig. 6 have been deposited to the Protein Data Bank under the accession codes EMD-3732 (non-rotated), EMD-3734 (first class with rotated 1 state, A/A and P/E tRNA), EMD-3735 (second class with rotated 1 state, A/A and P/E tRNA) and EMD-3736 (class with rotated two state, A/P and P/E tRNA).

## Electronic supplementary material

Supplementary Information

Supplementary Data 1

Supplementary Data 2

Supplementary Data 3
