# Supplementary material for: Ubiquitination of stalled ribosome triggers ribosome-associated quality control
Source: Nat Commun. 2017 Jul 31;8:159. doi: 10.1038/s41467-017-00188-1 (PMC5534433; doi:10.1038/s41467-017-00188-1)
Supplement: Supplementary file 1 — Supplementary Information [file 41467_2017_188_MOESM1_ESM.pdf]

File Name: Supplementary Information

Descriptions: Supplementary FIgures

File Name: Supplementary Data 1

Descriptions: MS analysis revealed that peptides representing the arrested products derived from GFPR(CGN)12-FLAG-HIS3 reporter.

File Name: Supplementary Data 2

Descriptions: Ubiquitinated ribosome proteins detected in ribosomes co-purified with Rqt1or Not4

File Name: Supplementary Data 3

Descriptions: List of strains, plasmids, antibodies used in this study

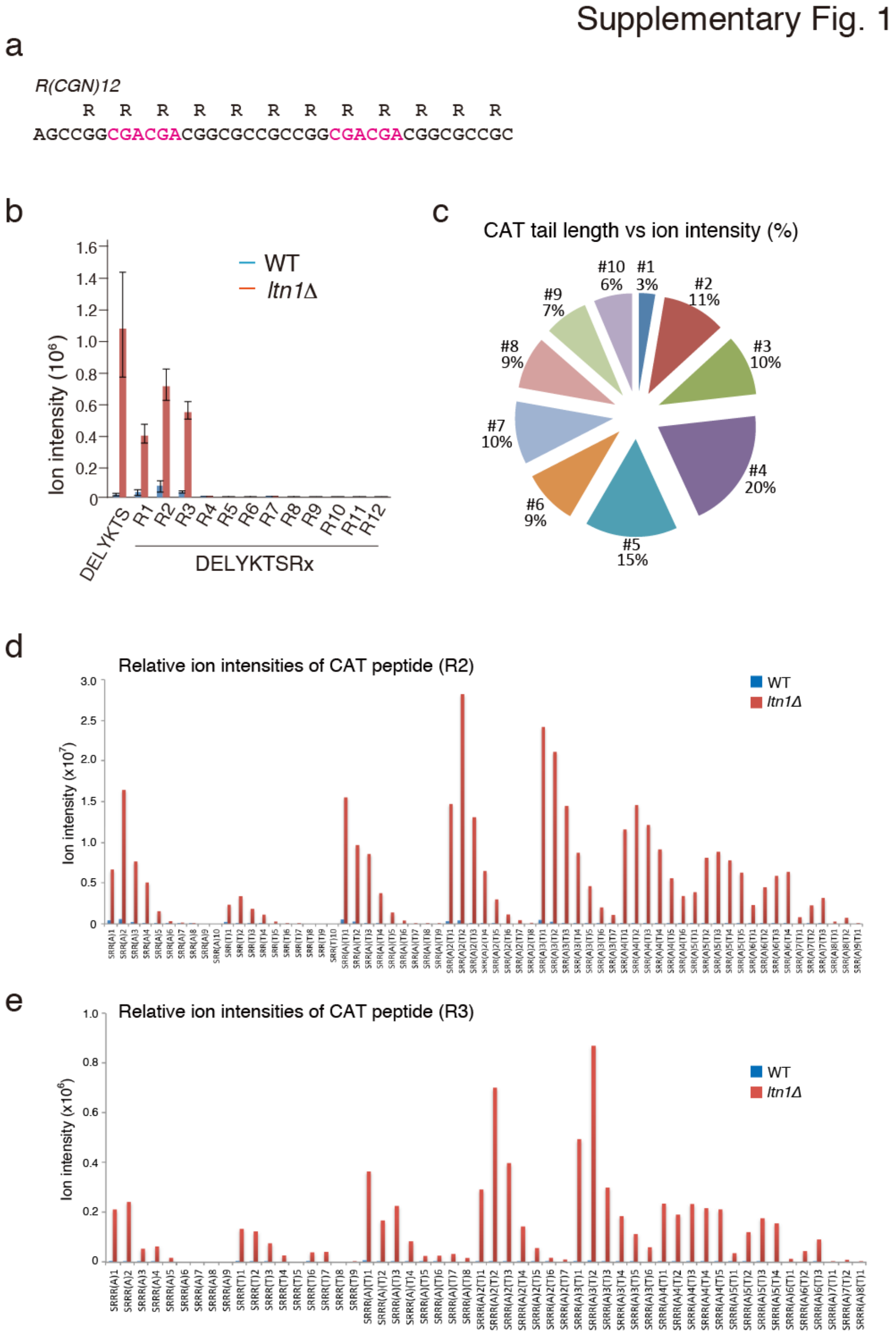

Supplementary Fig. 1

Mass spectrometry analysis of arrest products in *ltn1*Δ deleted background. (**a**) The sequence of R(CGN)12. CGA codons that are decoded by a single tRNA with inosine in the anticodon loop were shown in red. (**b**) The composition of the length of CAT-tail added to the arrest products derived from *GFP-R12-FLAG-HIS3* reporter in *ltn1*Δ mutant cells. The arrest products ended within three arginine residues in R12 arrest sequence. (**c**) The distribution of the length and composition of CAT-tail added to downstream of two arginine residues of the arrest products derived from *GFP-R12-FLAG-HIS3* reporter in *ltn1*Δ mutant cells. (**d-e**) The distribution of the length and composition of CAT-tail added to downstream of two or three arginine residues of the arrest products derived from *GFP-R12-FLAG-HIS3* reporter in *ltn1*Δ mutant cells.

Supplementary Fig. 2

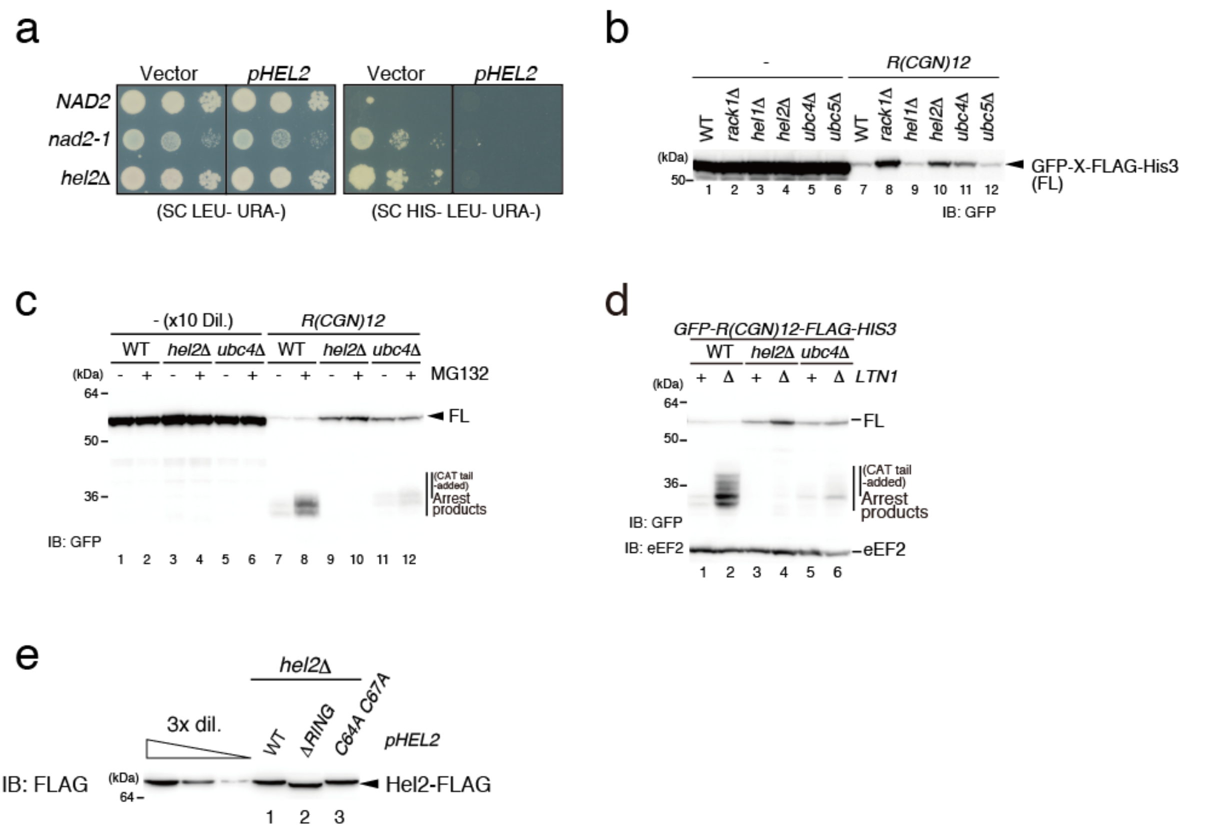

Supplementary Fig. 2

Hel2 and Ubc4 are required for RQC induced by a poly-arginine sequence. (**a**) The *GFP-R(CGN)12-FLAG-HIS3* reporter gene complements *his3*$^-$ in suppressor mutants. *NAD2*/*HEL2*, *nad2*/*hel2-1* suppressor mutant, or *hel2*Δ cells harboring the p416*CYC1p-GFP-R(CGN)12-FLAG-HIS3* reporter plasmid and the yCplac111 (vector) or p*GPDp-HEL2* plasmid were serially diluted and spotted onto SC-Leu-Ura or SC-His-Ura-Leu plate[21]. Direct sequencing identified a UAA nonsense mutation in the 178th codon of the *HEL2* gene of the *nad2-1* mutant. (**b**) Wild-type and indicated mutant cells that were transformed with the *GFP-FLAG-HIS3*(-) or *GFP-R(CGN)12-FLAG-HIS3(R(CGN)12)* reporter construct. Protein samples were prepared and analyzed by western blot analyses with anti-GFP antibody. (**c**) Reporter gene assay (*GFP-X-FLAG-HIS3*) in WT and indicated mutant cells. The products of reporter gene in the whole cell lysates were detected by anti-GFP antibody. The cells were treated with 0.5 mM MG132 (proteasome inhibitor) for 2 h. (**d**) Hel2 and Ubc4 ligases were crucial for induction of RQC. (**e**) Western blot analyses of the expression levels of the wild-type (WT) and RING domain Hel2/Rqt1 mutants.

Supplementary Fig. 3

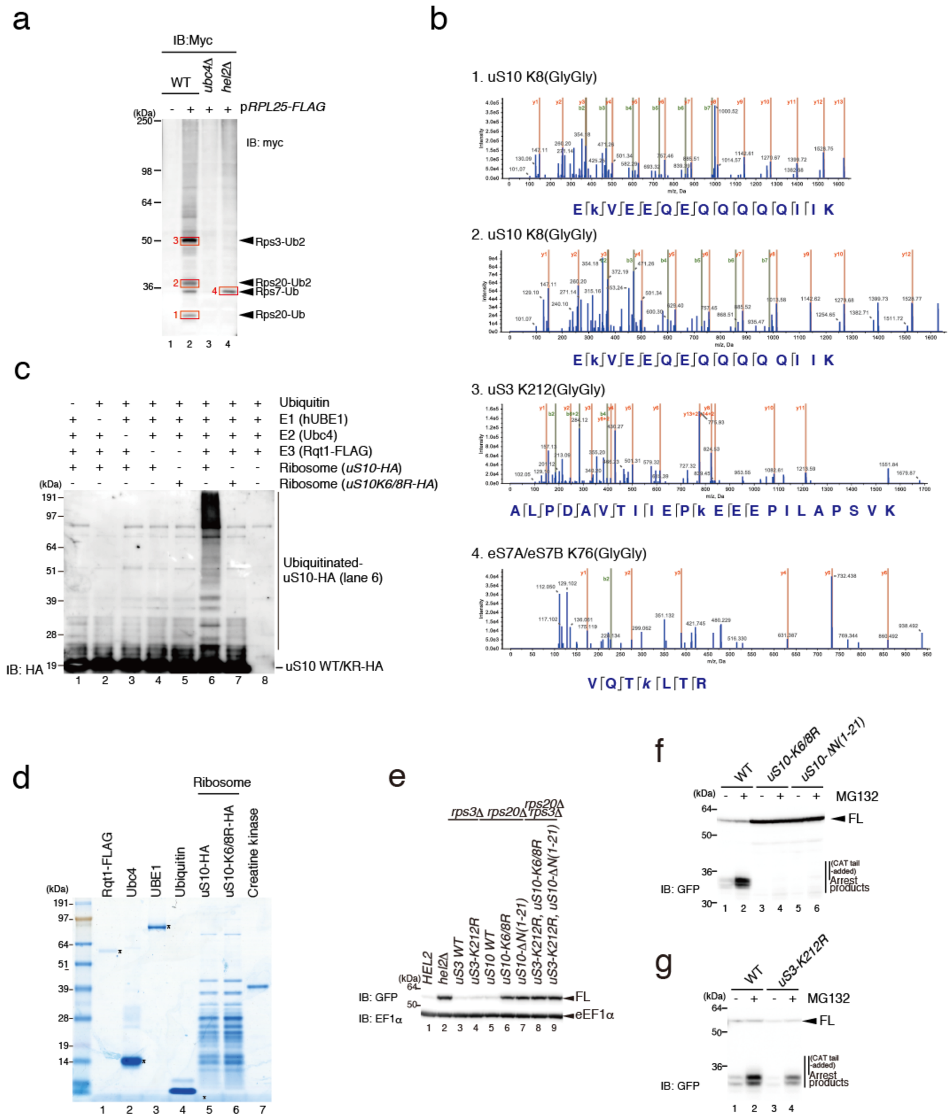

Supplementary Fig. 3

Mass spectrometry analyses of ubiquitinated ribosomal proteins. (**a**) Western blot analyses (anti-Myc) of FLAG-affinity purified samples from wild-type (WT) and the indicated mutant cells harboring the p*GPDp-RQT1* and p*CUP1*p-*MYC-UBI* plasmids, as well as the p*GPDp-RPL25(uL23)-FLAG* or control plasmid. To induce Myc-Ubi, the cells were cultured in the presence of 0.1 mM Cu^{2+} for 2 h. (**b**) MS/MS spectra of the identified ubiquitinated peptides isolated from the bands shown in (a). *b* and *y* fragment ions are denoted by green and red, respectively. The Lys residue of ubiquitination site, Lys-ε-GlyGly, is indicated by a small letter. (**c**) Rqt1 could ubiquitinate uS10 at K6/8 residues *in vitro*. The ubiquitinated uS10-HA was detected by western blot using anti-HA antibodies. (**d**) CBB stain of components for the *in vitro* ubiquitination reaction. (**e**) The protein levels of GFP-R(CGN)12-FLAG-His3 were significantly increased in *rqt1*Δ or uS10-K6/8R, uS10-ΔN(1-21) mutant cells. (**f**) Rqt1-dependent ubiquitination of uS10 was crucial for RQC. (**g**) Rqt1-dependent ubiquitination of uS3 was not crucial for RQC. (**f-g**) Western blot analyses of the indicated cells harboring *GFP-R(CGN)12-FLAG-HIS3* with anti-GFP antibody. Where indicated, the cells were treated with 0.5 mM MG132 for 2 h. FL, full-length.

Supplementary Fig. 4

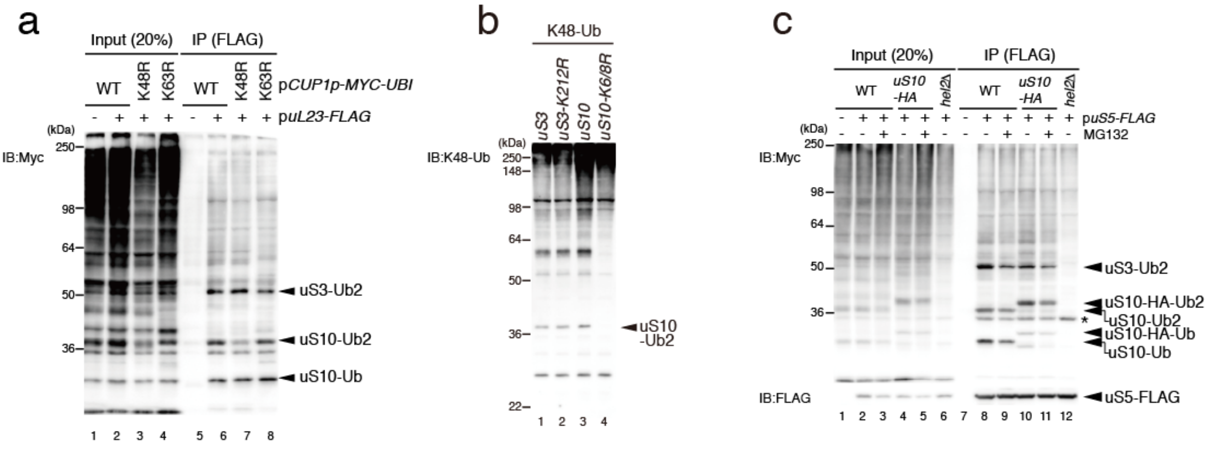

Supplementary Fig. 4

The uS10 ubiquitination is K48-linked, but not degradation signal. (**a**) The ubiquitination of uS10 was mainly K48-linked. Samples were prepared from wild-type cells harboring p*CUP1*p-*MYC-UBI* (WT), p*CUP1*p-*MYC-UBI-K48R* (K48R) or p*CUP1*p-*MYC-UBI-K63R* (K63R). (**b**) The detection of K48-linked ubiquitinated uS10 and uS3 proteins by Western blot analysis with K48-linkage specific polyubiquitin (D9D5) rabbit mAb (#8081, Cell signaling, USA). (**c**) The levels of ubiquitinated uS10 or uS3 were not increased by the inhibition of proteasome. (**a-c**) The ribosomes were affinity purified by uL23-Flag-tag, and ubiquitinated proteins were detected by indicated antibody.

Supplementary Fig. 5

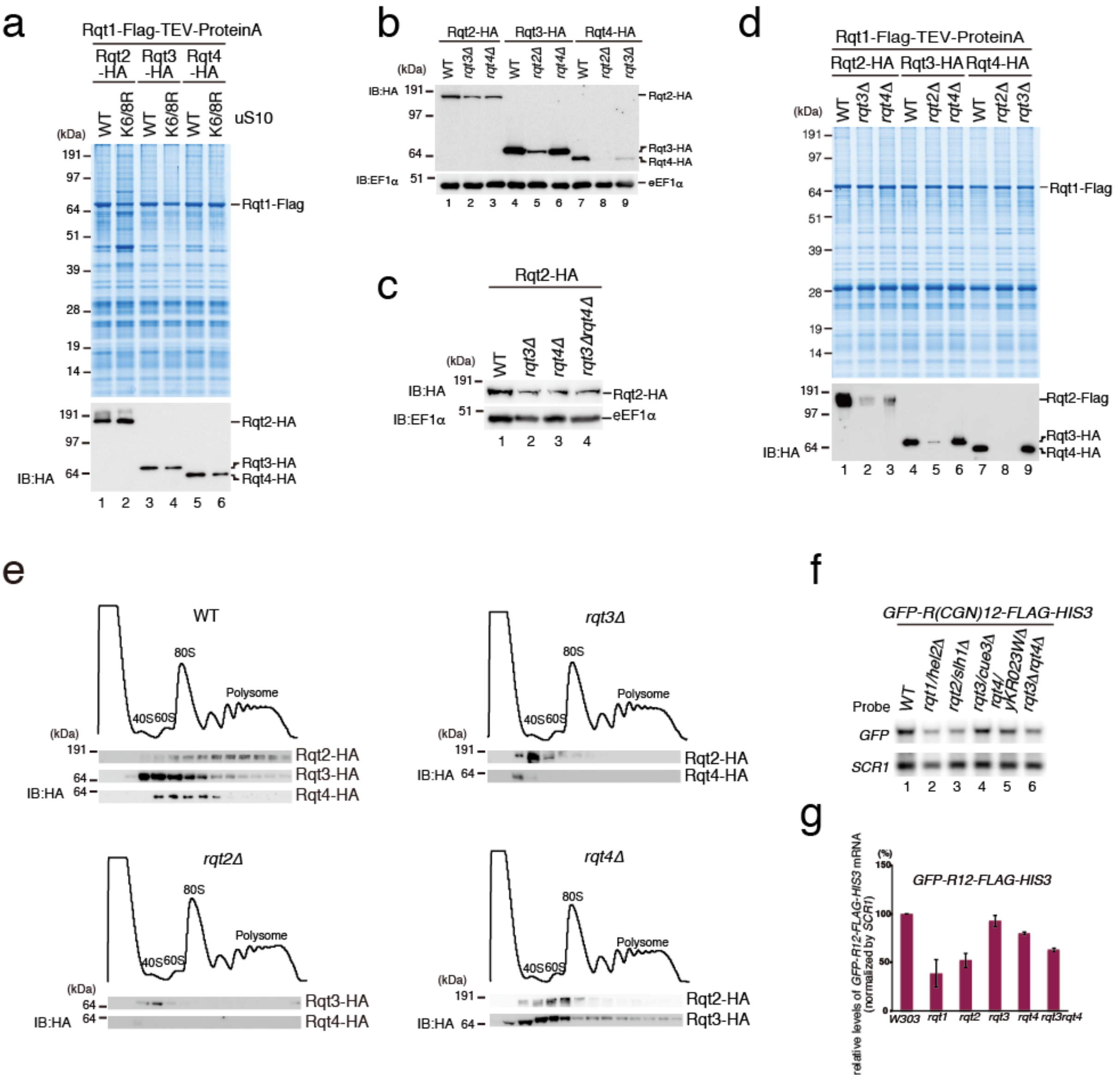

Supplementary Fig. 5

The relationship among Rqt factors (**a**) The ubiquitination of uS10 is dispensable for the association of Rqt2, Rqt3 and Rqt4 in Rqt1-ribosome complex. (**b**) The expression levels of HA-tagged Rqt proteins expressed from endogenous tagged alleles in *rqt* mutant cells. (**c**) The expression levels of Rqt2-HA expressed from endogenous tagged alleles were slightly decreased in *rqt3* or *rqt4, rqt3rqt4* mutant cells. (**d**) Interdependency of Rqt2-3-4 factors on the association with Rqt1-ribosomes. **(e)** The distribution of Rqt2-4 proteins in polysome profiles of indicated mutants. (**f**) Northern blot analysis of mRNA levels of *GFP-R(CGN)12-HIS3* reporter in *rqt* mutant cells. (**g**) The quantification of mRNA levels of *GFP-R(CGN)12-HIS3* reporter in *rqt* mutant cells.

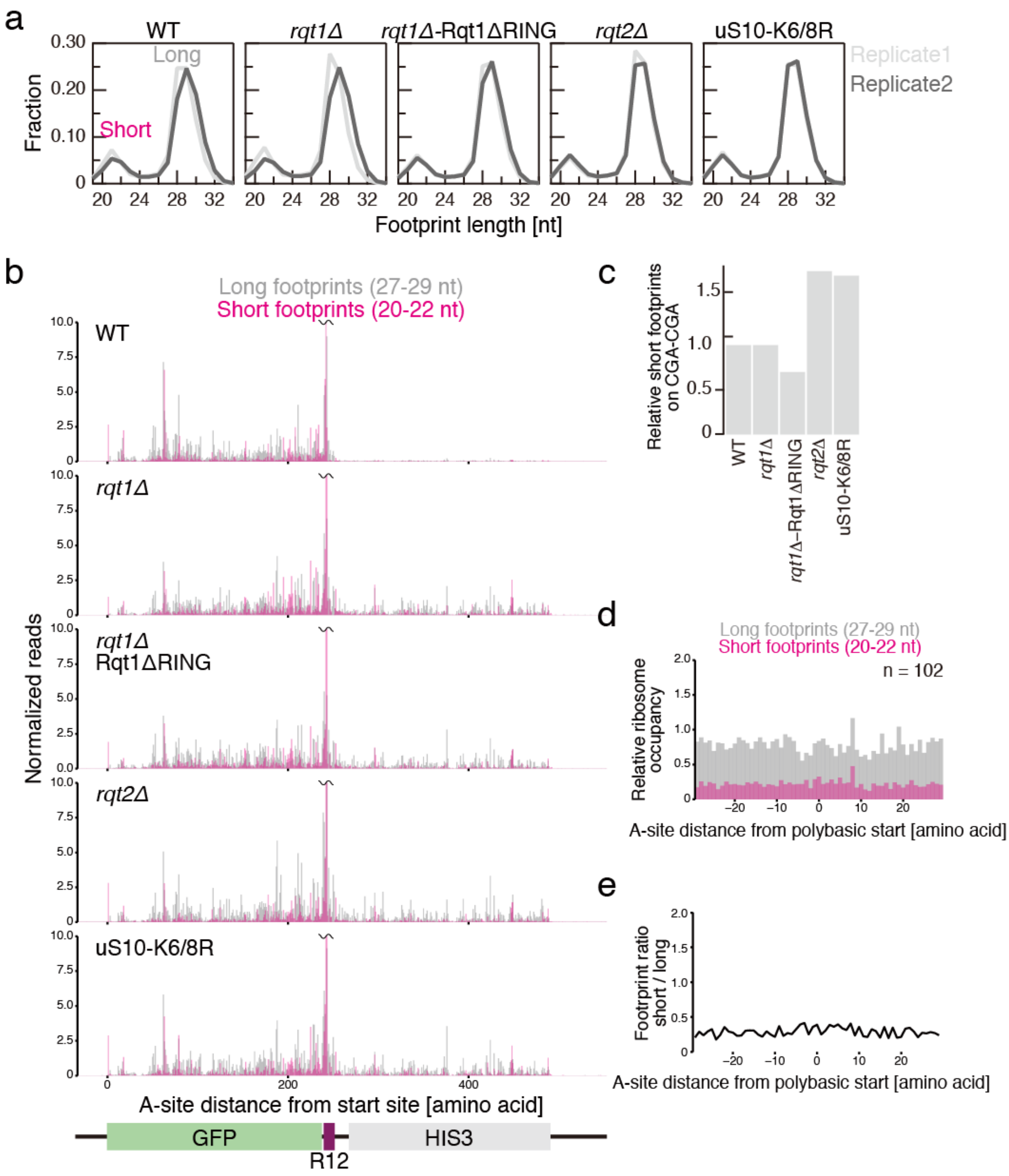

Supplementary Fig. 6

Stalled ribosomes in a rotated state conformation are targets for RQC. (**a**) Distribution of length of footprints in wild type and the mutants. (**b**) Mapping of short (20-22 nt) and long (27-29 nt) footprints along entire reporter mRNAs. (**c**) Ribosome occupancy of *HIS3* region, downstream of R12, was recovered in the Rqt mutants. (**d**) Long and short footprints were accumulated at 8 amino acid downstream from polybasic tract start site. **(e)** The ratio between short and long footprints is not changed by polybasic tract.

Supplementary Fig. 7

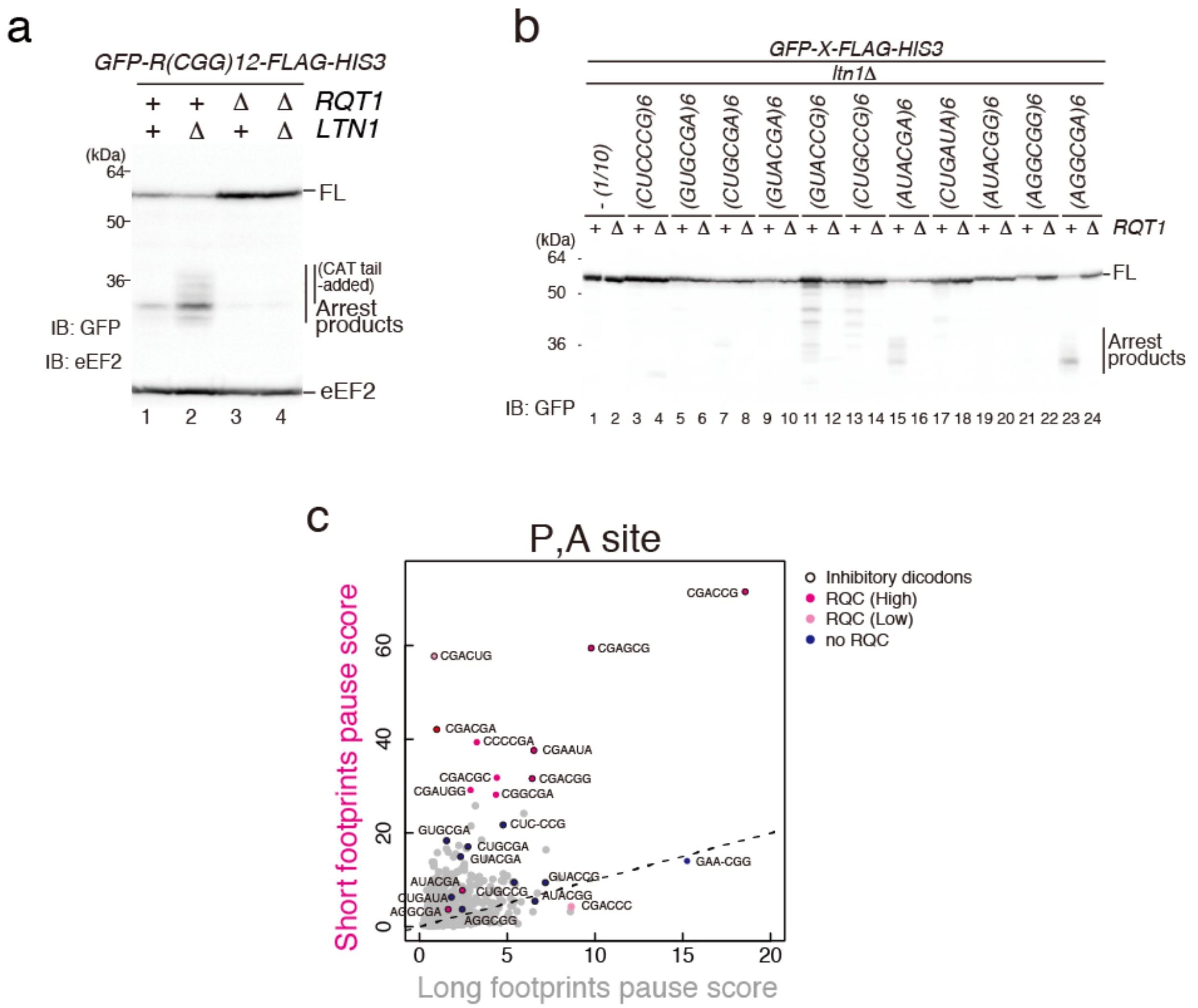

Supplementary Fig. 7

Inhibitory di-codons are the feature of translation arrest, but not RQC targets (**a**) Rqt1 was required for RQC by the repeats of CGG codon. (**b**) In 17 inhibitory di-codons that were identified by Grayhack and co-workers, six di-codons were included in top 10 di-codons that accumulated short footprints, and induced RQC. In other 11 di-codons, only two di-codons, AUACGA and AGGCGA induced RQC. **(c)** The accumulation of short footprints is feature of RQC targets, but not long footprints, which is the feature of general ribosome stalling. It was shown that translation inhibitory 17 di-codons (black frame bots) are correlated with the long footprints accumulation. 8 of them induced RQC (magenta circle), but others not (blue circle). CGACCC accumulated long footprints and moderately induced RQC (light magenta circle).

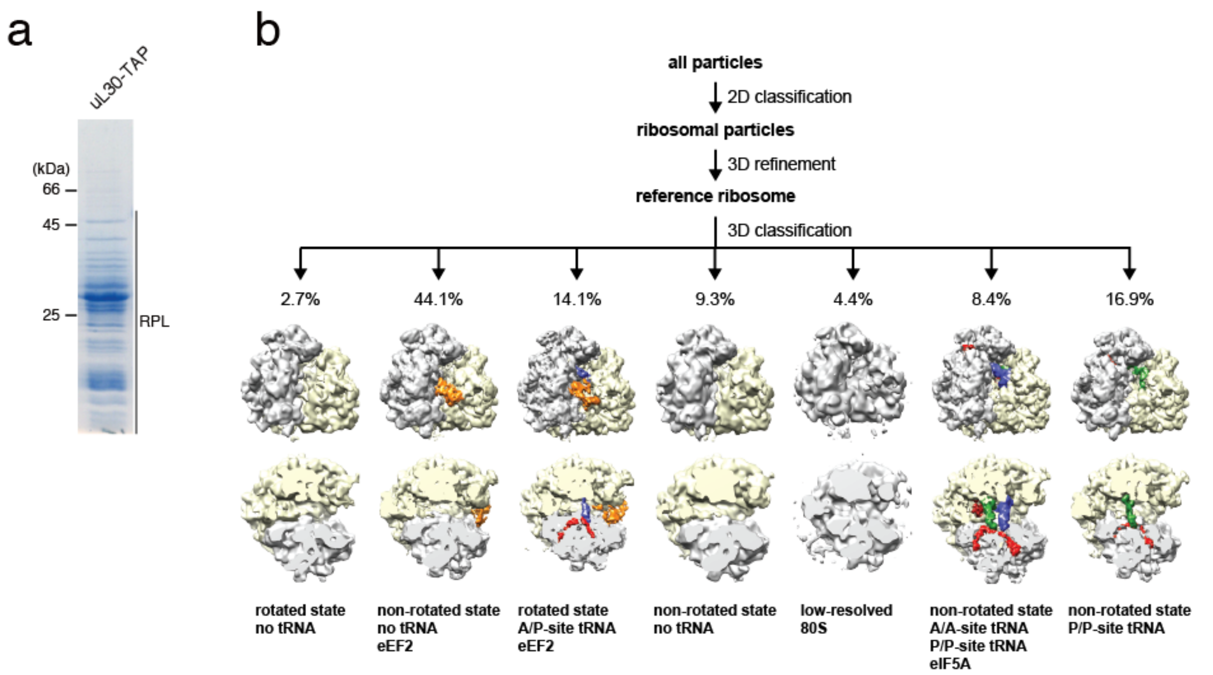

Supplementary Fig. 8

Cryo-EM analysis of uL30 pull out ribosome (**a**) CBB stained SDS gel and Amidoblack stained blot of pullouts from uL30-TAP strains. (**b**) Particle sorting scheme of the uL30-ribosome pull-out sorted into 7 classes. In total, the dataset contained 60.6% unprogrammed ribosomes (partially "sleeping" ribosomes with eEF2 bound), 25.3% classical state ribosomes, and 14.1% rotated state ribosomes with A/P-site tRNA and eEF2.

Supplementary Fig. 9

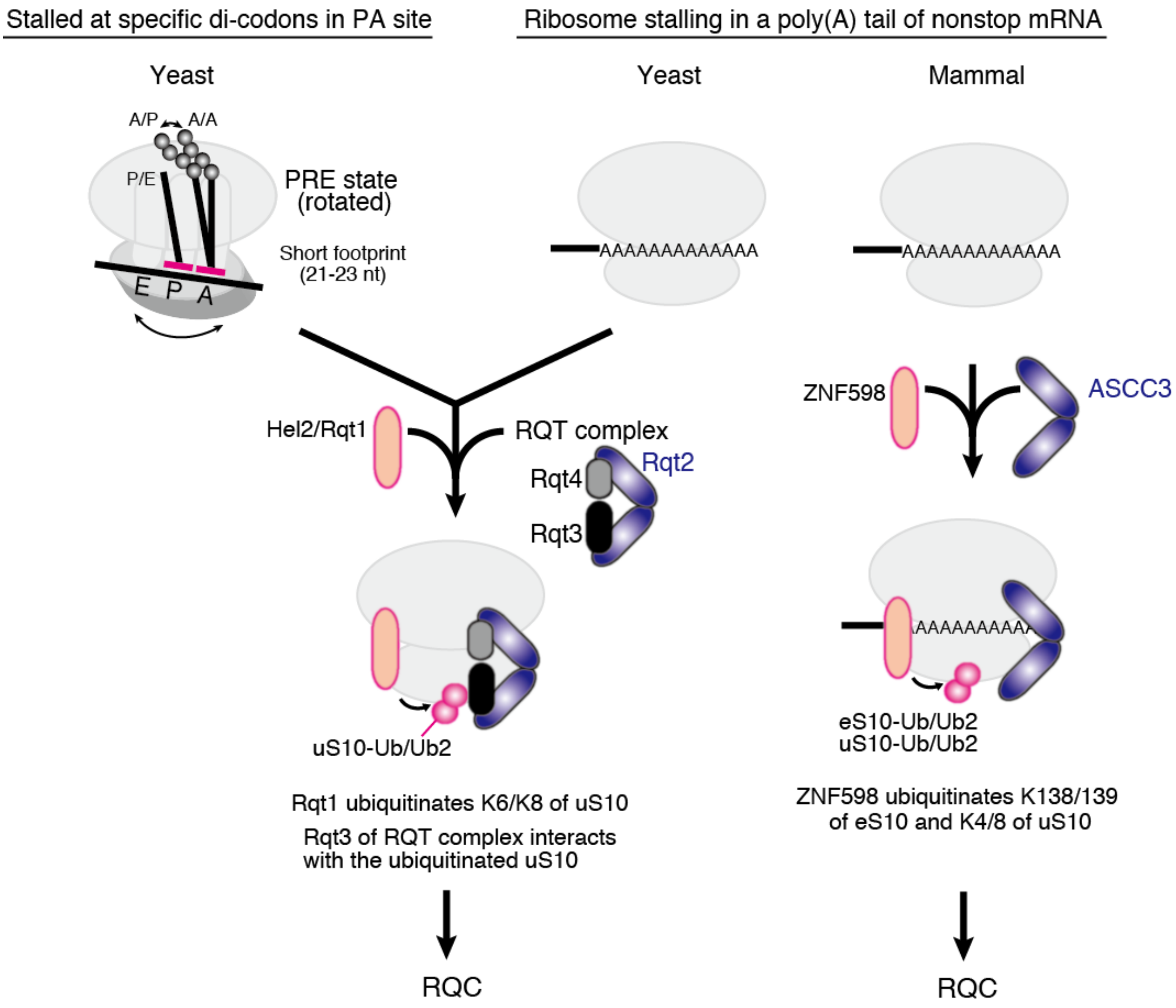

Supplementary Fig. 9

A model for the roles of Rqt factors in the ribosome quality control triggering step. **(Left, in yeast)** Rqt1 binds to the rotated ribosome with hybrid tRNAs and ubiquitinates uS10 at K6/8 due to the wobble decoding. Rqt complex is involved in the dissociation of the stalled 80S ribosome into 40S and 60S subunits via the interaction of Rqt3 with ubiquitinated uS10. An E3 ligase Ltn1 ubiquitinates the peptidyl-tRNA on the 60S subunit. The ubiquitinated peptidyl-tRNA is extracted by Cdc48 with co-factors, and degraded by the proteasome. **(Middle, in yeast)** Rqt1 binds to the stalled ribosome translating poly(A) sequence. Rqt complex is involved in the dissociation of the stalled 80S ribosome into 40S and 60S subunits via the interaction of Rqt3 with ubiquitinated uS10. **(Right, in mammal)** ZNF598 binds to the stalled ribosome translating poly(A) sequence, and ubiquitinates uS10 and eS10[33,34]. ASCC3 is involved in the dissociation of the stalled 80S ribosome into 40S and 60S subunits via the interaction with putative factor that recognizes the ubiquitinated uS10 or sS10[33,34].

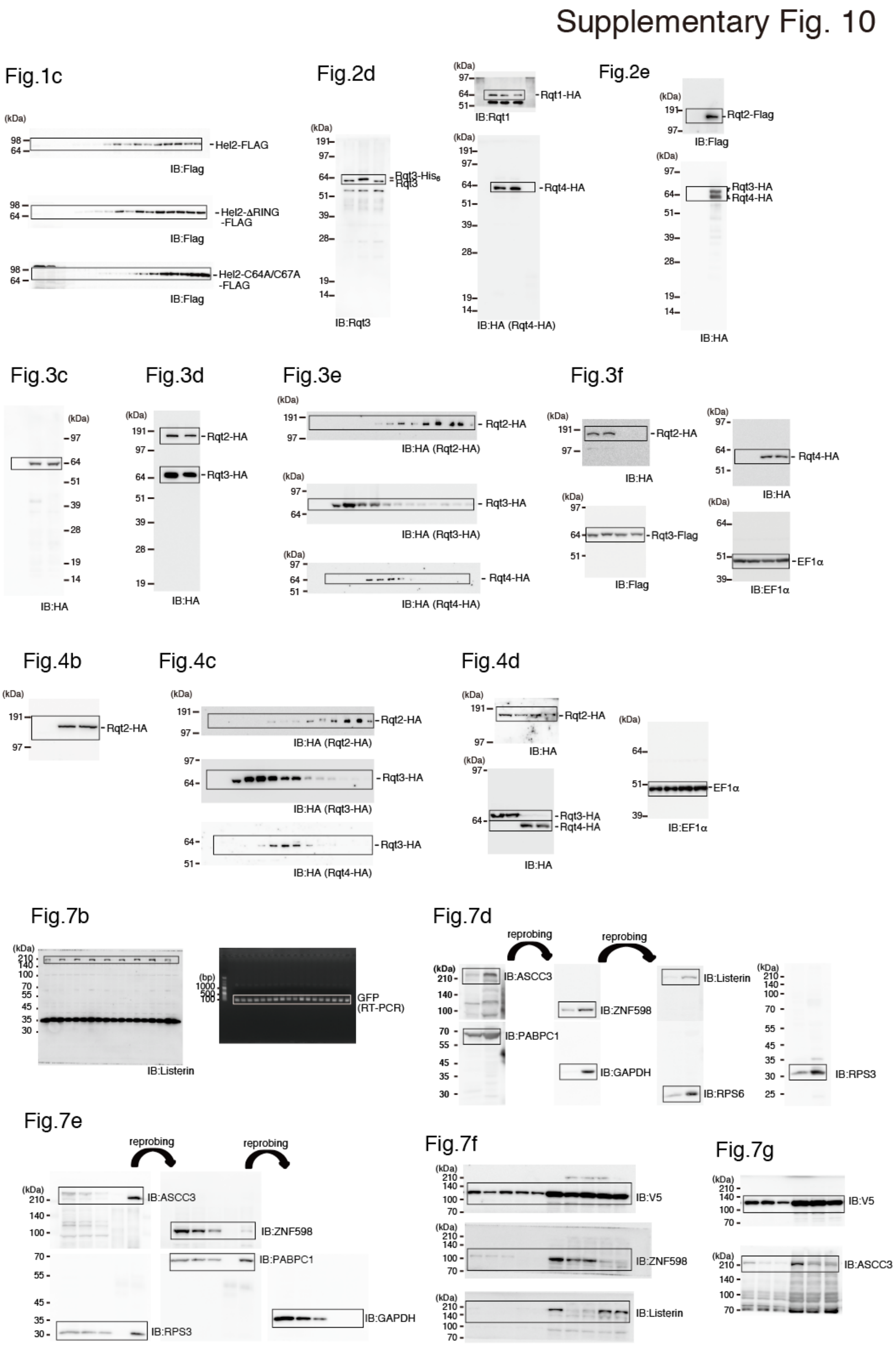

Supplementary Fig. 10

Uncropped Western blots related to Figure 1, 2, 3, 4 and 7.
